# The MIS5 Pietersburg at ‘28’ Bushman Rock Shelter, Limpopo Province, South Africa

**DOI:** 10.1371/journal.pone.0202853

**Published:** 2018-10-10

**Authors:** Guillaume Porraz, Aurore Val, Chantal Tribolo, Norbert Mercier, Paloma de la Peña, Magnus M. Haaland, Marina Igreja, Christopher E. Miller, Viola C. Schmid

**Affiliations:** 1 CNRS, UMR 7041, ArScAn-AnTET, Université Paris Ouest Nanterre La Défense, Paris, France; 2 Evolutionary Studies Institute, University of the Witwatersrand, Johannesburg, South Africa; 3 Ditsong National Museum of Natural History, Pretoria, South Africa; 4 CNRS, UMR 5060, IRAMAT-CRP2A, CNRS-Université Bordeaux Montaigne, Bordeaux, France; 5 Department of Archaeology, History, Cultural Studies and Religion, University of Bergen, Bergen, Norway; 6 Centre for Early Sapience Behaviour (SapienCE), University of Bergen, Bergen, Norway; 7 University of Porto, Porto, Portugal; 8 Institute for Archaeological Sciences & Senckenberg Centre for Human Evolution and Palaeoenvironment, Universität Tübingen, Tübingen, Germany; 9 Abteilung für Ältere Urgeschichte und Quartärökologie, Universität Tübingen, Tübingen, Germany; Max Planck Institute for the Science of Human History, GERMANY

## Abstract

In the past few decades, a diverse array of research has emphasized the precocity of technically advanced and symbolic practices occurring during the southern African Middle Stone Age. However, uncertainties regarding the regional chrono-cultural framework constrain models and identification of the cultural and ecological mechanisms triggering the development of such early innovative behaviours. Here, we present new results and a refined chronology for the Pietersburg, a techno-complex initially defined in the late 1920’s, which has disappeared from the literature since the 1980’s. We base our revision of this techno-complex on ongoing excavations at Bushman Rock Shelter (BRS) in Limpopo Province, South Africa, where two Pietersburg phases (an upper phase called ‘21’ and a lower phase called ‘28’) are recognized. Our analysis focuses on the ‘28’ phase, characterized by a knapping strategy based on Levallois and semi-prismatic laminar reduction systems and typified by the presence of end-scrapers. Luminescence chronology provides two sets of ages for the upper and lower Pietersburg of BRS, dated respectively to 73±6ka and 75±6ka on quartz and to 91±10ka and 97±10ka on feldspar, firmly positioning this industry within MIS5. Comparisons with other published lithic assemblages show technological differences between the Pietersburg from BRS and other southern African MIS5 traditions, especially those from the Western and Eastern Cape. We argue that, at least for part of MIS5, human populations in South Africa were regionally differentiated, a process that most likely impacted the way groups were territorially and socially organized. Nonetheless, comparisons between MIS5 assemblages also indicate some typological similarities, suggesting some degree of connection between human groups, which shared similar innovations but manipulated them in different ways. We pay particular attention to the end-scrapers from BRS, which represent thus far the earliest documented wide adoption of such tool-type and provide further evidence for the innovative processes characterizing southern Africa from the MIS5 onwards.

## Background to the Pietersburg legacy

Over the last three decades, archaeological research and excavations in South Africa have largely contributed to expand our knowledge on the way Anatomically Modern Humans (AMHs) were territorially, technologically and socio-economically organized. Such studies have emphasized early manifestations of advanced technologies and symbolically-mediated behaviours during the Middle Stone Age (MSA). Amongst others, the application of pressure flaking to shape stone tools [1,2], use of heat treatment to alter rock properties [3,4], fabrication of beads [5,6] and deliberate engravings [7–10] are regarded as relevant evolutionary proxies within the history of AMHs. Most of these archaeological discoveries have been found in technological contexts that relate to the Still Bay and Howiesons Poort. These two techno-complexes benefit from a large scientific exposure, though their definition is still disputed. Indeed, while on the one hand the spatial and technological homogeneity of the Still Bay has recently been questioned [2,11–14], the Howiesons Poort, on the other hand, still presents a chronology [15–18] and technological subdivision into sub-phases [11,19–22] that require clarification.

Debates surrounding the Still Bay and the Howiesons Poort mask some broader issues, which reflect the uncertainties currently characterizing the chrono-cultural framework of the southern African MSA. Most recent works have focused on specific behavioural proxies and particular techno-complexes, neglecting long archaeological sequences that provide local scenarios of technical adaptations and transformations. In that perspective, the Western and Eastern Cape Provinces represent a noticeable exception (e.g., [11, 23–26]). However, the archaeological record from those regions only provides data for a single ecological biome amongst the many other ones that are found in South Africa. Contrastingly, past researchers were more concerned by the need to study long sequences and elaborate preliminary syntheses on broader geographical scales (e.g., [27–32]).

Of interest are the sites and syntheses from the former Transvaal region, in particular from today’s Limpopo and Gauteng Provinces. In a period between the 1940’s and the 1960’s, many sites were excavated (e.g., Mwulu’s Cave, Heuningneskrans, Rainbow Cave, Olieboomspoort Shelter, Aasvöelkop) but, since then, work conducted in the area has virtually been inexistent (but see [33,34]). Amongst the set of excavated sites, Cave of Hearths and Bushman Rock Shelter (BRS) represent two sequences that serve as references for the MSA of that region. Both sites provide long archaeological sequences that are said to contain Pietersburg industries, a techno-complex initially recognized in the late 1920’s and described at that time by Goodwin and van Riet Lowe as a MSA variation that needed clarification [35]. Van Riet Lowe [36] considered it in the 1940’s as a definite “industrial” MSA complex and Clark [37] listed it as one MSA variant. However, it was only in 1957 that the Pietersburg was formally defined for the first time by R. Mason, using the sequence of Cave of Hearths: « *The interpretation of the data depends on the six superposed Middle Stone Age beds in the Cave of Hearths*. *These beds yielded seven industries that provide a remarkably clear picture of the nature and evolution of the MSA Pietersburg Culture from an early stage to a late stage*” ([38]: 120). Mason [38] applied an analysis based on lithic attributes and individualized three distinct phases (i.e., Early, Middle and Late) that reflect various proportions in blades and triangular flakes productions as well as differences in formal tool composition (e.g. presence/absence of end-scrapers and of bifacial pieces). In his synthesis, Sampson ([29]: 66) regards the Pietersburg as “*an entirely valid and acceptable term*, *supported by at least 10 large unselected samples*”.

**Fig 1:**
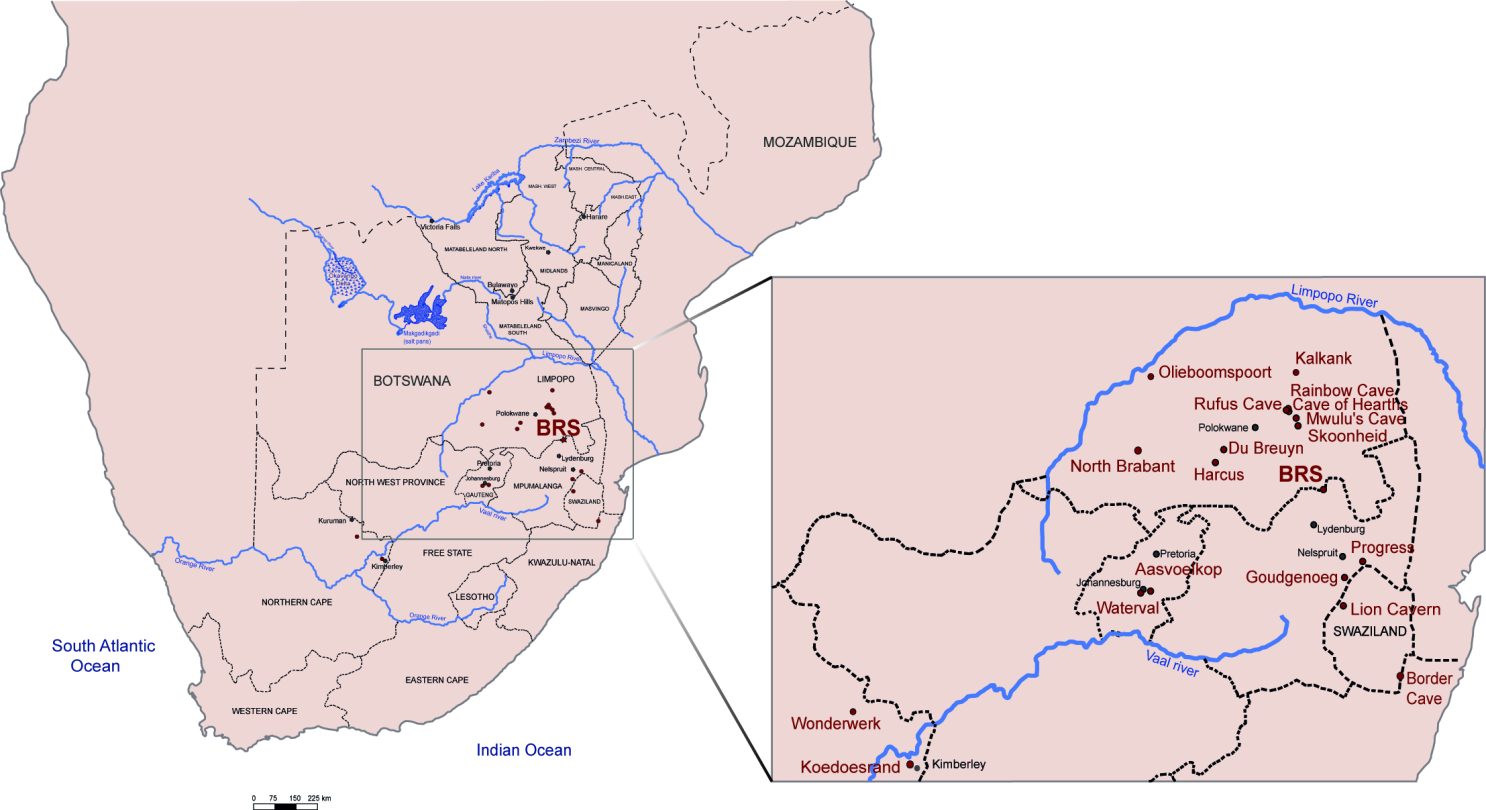
Location of Bushman Rock Shelter (Limpopo Province, South Africa) and other MSA sites with occupations assigned to the Pietersburg.

Besides Cave of Hearths and BRS, the Pietersburg has been identified at sites such as Mwulu’s Cave [39], Olieboomspoort Shelter [38], Rainbow Cave [40], Border Cave ([41,42] but see [38]) and several others (see Fig 1). The present distribution of Pietersburg sites suggests that this technological tradition may represent a regional expression occurring in the interior of South Africa, south of the Limpopo River [43]. However, most of the Pietersburg lithic assemblages lack appropriate descriptions and very little is known regarding their chronology. Apart from a set of radiocarbon dates falling beyond the range of this method (see [44]), the only dates presently available come from the site of Border Cave, which provides ESR ages bracketed between 227 and 82 ka BP [45,46] (and see [47] for Wonderwerk). This lack of accurate chronology, together with the absence of clear technological and typological descriptions, contributes to explain why the Pietersburg progressively disappeared from the literature from the 1980’s onwards. Recently, Lombard *et al*. [48] listed the Pietersburg as a variant of the Mossel Bay (or MSA II) and Klasies River (or MSA I) techno-complexes.

In this paper, we provide new data on the chronology and lithic technology of the upper MSA deposits of BRS. These deposits record two techno-typological phases that are both reminiscent of the Pietersburg (following [38]) and that we individualize as the upper phase ‘21’ and the lower phase ‘28’. The upper Pietersburg of BRS is dated to 73±6ka (OSL quartz) and 91±10ka (OSL feldspar) while the lower Pietersburg is dated to 75±6ka (OSL quartz) and 97±10ka (OSL feldspar). We focus our technological and functional study on the lower phase, which documents a tradition characterized by Levallois and semi-prismatic laminar reduction strategies and by the manufacture of end-scrapers and side-scrapers. We compare this technology with sub-contemporaneous assemblages and conclude that groups with different technological traditions were coexisting in southern Africa during the MIS5. We argue that the cultural mosaic present during the MIS5 provided a favorable context for the florescence of innovative behaviours from around 100ka. We emphasize the importance of developing long regional cultural sequences and the need to position these technological traditions within a broader cultural and geographical perspective.

## Bushman Rock Shelter: Brief presentation of past and ongoing excavations

BRS is located on the north-eastern fringe of the Drakensberg mountain range, in the Limpopo Province, at an altitude of ca. 950 masl. The shelter is positioned at the confluence of the intermittent Molapong River with the perennial Ohrigstad River, flowing northeast. The shelter is located nearby the dramatic Blyde River Canyon, a physical and ecological frontier separating the Highveld from the Lowveld.

The shelter faces south and opens up in the dolomitic Malmani formation, offering a 900 m^2^ protected space. First excavations at the site occurred in 1965 under the supervision of A.W. Louw. The main investigation and record-keeping, however, originates from Prof. J.F. Eloff, who started his excavation in 1967 for a duration of 10 successive field seasons [49–51]. Eloff’s work at the site exposed up to 7.5 m thick deposits and revealed a continuous archaeological sequence characterized by a fairly high stratigraphic resolution and good organic preservation. Nevertheless, and with the exception of I. Plug’s work [50,52–54], very little was published and the archaeological record at the site remains therefore largely unknown.

Despite the quality of Eloff’s excavations [51], there was a need for clarification before further publications. In 2014, two of us (G.P. & A.V.–permit ID2475 granted by the South African Heritage Resources Agency) started leading new excavations at the site with the following four main field objectives: (1) to record Eloff’s stratigraphic profiles, (2) to characterize the site formation processes, (3) to establish a chronology, and (4) to sample the deposits. To avoid any confusion with Eloff’s excavation, we developed our own recording system. While Eloff used Arabic numbers to distinguish his layers (starting from layer 1 at the top until layer 107 at the base), we followed a system that subdivided the sequence, firstly into stratigraphic blocks, each individualized by a letter (starting from A at the top of the sequence) and secondly, within each block, into Stratigraphic Units (SUs) by giving two syllabled names that follow the alphabetical order.

The sequence of BRS can be subdivided into two main chrono-cultural phases that represent Later Stone Age (LSA) and MSA occupations. While the LSA sequence (layers 2 to 16) is fairly well dated with occupations bracketed between ca. 10,900 and 15,000 cal BP, no secure dating has yet been published for the MSA (from layer 19 to 107). The LSA deposits are separated from the MSA ones by a clear hiatus marked by a thick accumulation of dolomite rocks (layers 17 and 18). This accumulation appears to be the cause of some mixing in the archaeological material that has led to the hypothesis [49] -rejected since [51,54]- of a transitional phase from the MSA to the LSA.

The MSA of BRS was initially assigned to the Pietersburg by Eloff (“*a phase which seems to represent a south-eastern expression of the Pietersburg industrial complex*” [49]: 60), who mentioned similarities with the lithic assemblages of Cave of Hearths. In the early 1980’s, Singer and Wymer [31] suggested that MSA artifacts of BRS might equate with those from the MSA I and II of Klasies River Mouth. In T. Volman’s synthesis [32], the MSA sequence of BRS is said to contain MSA 1 (layers 107–31), MSA 2 (layers 30–19) and MSA 3 (layers 18–15) occupations. More recently, Badenhorst and Plug [50] proposed that the MSA occupations from layers 22 to 30 may be contemporaneous with post-HP assemblages. Following preliminary observations of the lithic collections from the upper MSA deposits (that we associate with layers 19 to 40), we distinguished three techno-typological phases on the base of raw materials, blank characteristics and typological corpus. To avoid preliminary assumptions, we temporally called these phases ‘21’, ‘28’ and ‘36’ (following their eponymous layers from Eloff’s excavation).

**Fig 2:**
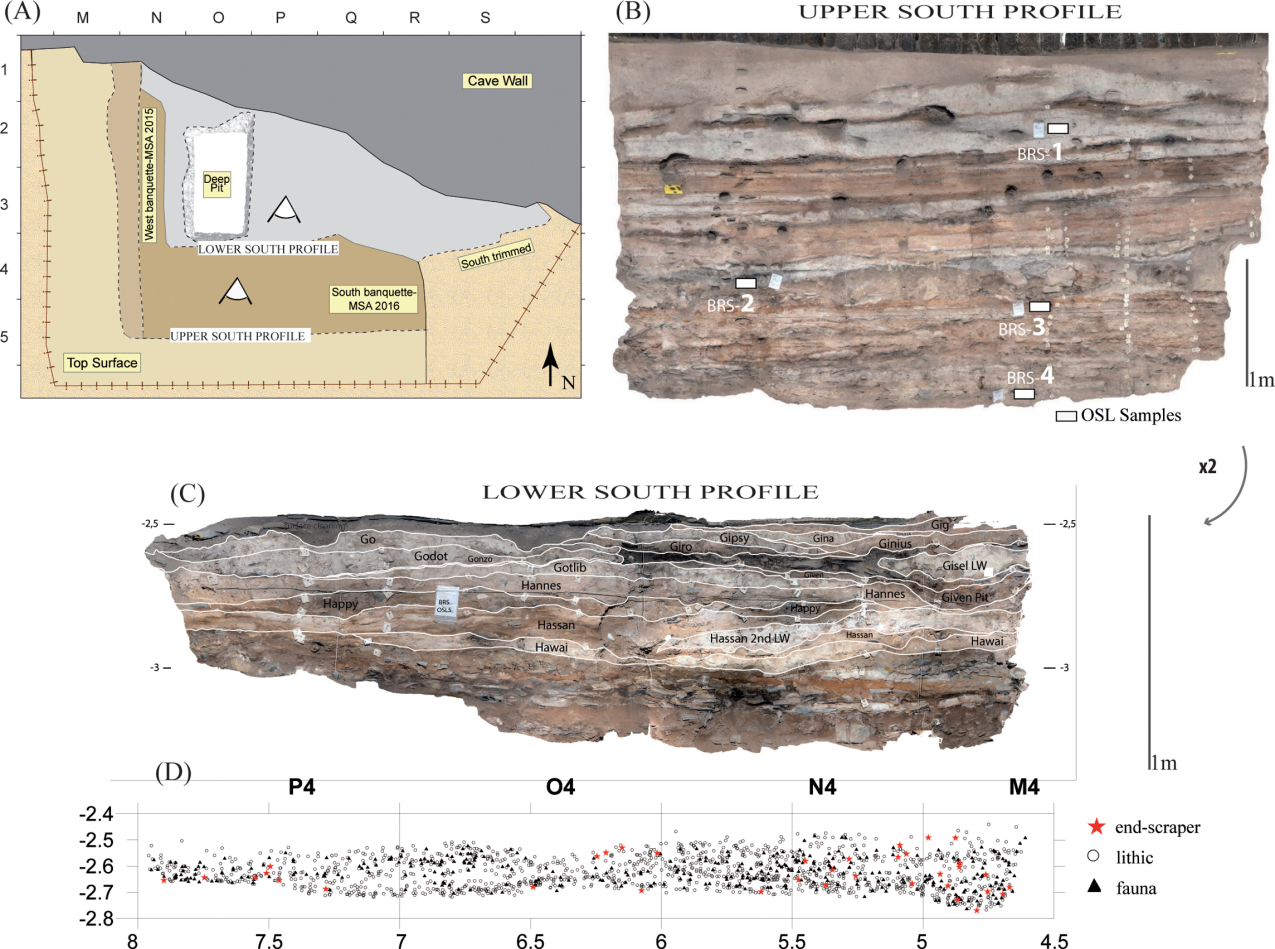
(A) Planimetry of the excavation area at Bushman Rock Shelter, (B) ortho-photographic image of the Upper South Section with location of the four OSL samples (BRS1-4) and (C) labelled MSA Stratigraphic Units excavated in 2016 and 2017 with (D) projection of the plotted material from the 2016 excavation.

Because of the geometry of the deposits (directly inherited from past excavations), we started our MSA excavation on layer 27; thus, so far, renewed field data only concern the so-called phase ‘28’. We started our excavation in 2015 and opened two separate areas (the east and the west ‘*banquettes’*, or steps) to secure and prepare our main sector (the south ‘*banquette’*) that we started excavating in 2016 (Fig 2).

For the present paper, we focus primarily on the material from the main sector i.e., south ‘*banquette’*, which has been opened on a surface of ca. 3.5 m^2^ (4 m x 0.7 m). Following available field notes as well as the numerous labels still tagged in the stratigraphy, we were able to get a good understanding of Eloff’s excavation, though we recognized a somewhat different stratigraphic succession. Unlike Eloff, who identified layers that were laterally continuous across the southern profile, we subdivided the sequence into two parts that are formed by the western SUs (SUs starting with the prefix ‘Gi-‘) and the eastern ones (those starting with the prefix ‘Go-‘) (Fig 2). These deposits present a general dome shape and both groups of SUs pinch in the middle of our excavated area. We stopped our 2016 excavation on top of layer 31 (SU ‘Hannes’), which is laterally continuous from west to east and which is presently considered as the top of our technological phase ‘36’.

The 2016 excavation allowed us to plot 1871 objects, including 1441 lithic pieces (≥20 mm and all cores and tools fragments recognized during excavation), 39 ochre pieces (≥20 mm) and 373 faunal remains (≥20 mm and all identifiable elements) (Fig 2). Charcoal fragments were all collected during dry sieving using 3 and 1 mm mesh, while other macrobotanical remains such as seeds and corms, when noticed during excavation, were removed and packed separately. During excavation, we strictly respect the sediments’ characteristics (i.e., grain size, texture and colour) to define our SUs. Within each SU, we individualize several ‘*décapages’* (or surfaces) [26], which all receive a number (e.g., ‘Gisel 1’, ‘Gisel 2’). A *décapage* represents an archaeological sub-horizontal plane that is defined by, at least, the base of three archaeological objects. Every *décapage* is photographed and geo-referenced following a photogrammetric protocol that links it with the total station database. In addition to SUs, we individualize all features and lenses represented by sedimentary events that do not extend beyond 1 m^2^. These features are named according to their nature and relative chronology (e.g., ‘Gabi Hearth within’, ‘Gisel Pit from’).

## The phase ‘28’ at Bushman Rock Shelter

We previously reported on the techno-typological phase ‘21’ in Porraz et al. [51] and proposed that these lithic assemblages were reminiscent of the Late Pietersburg. By contrast to ‘21’, we distinguished the techno-typological phase ‘28’ mostly based on its remarkably rich amount of end-scrapers. End-scrapers occur from layers 24 to 31 and overlap with bifacially shaped tools in layers 24 and 25. However, it is presently unclear whether the association of end-scrapers with bifacially shaped tools illustrates a continuous typological change or, rather, reflects some post-depositional mixing (within the deposits or during past excavations).

For the present paper, we focus on the layers 27 to 30 of the phase ‘28’, which form *ca*. 50 cm thick deposits. Eloff’s collection revealed many peculiarities. First of all, this phase has yielded beads [54], including five made of ostrich eggshell (OES) [50] and one of giant snail (*Achatina* sp.) shell. These beads originate from the layer 27(i) and 28a, all from square J3. In South Africa, the earliest beads documented are made of marine shells, namely *Nassarius kraussanius*, and come from the Still Bay layers of Blombos Cave [5]; they date back to 72,000 years BP. The oldest occurrence currently known of beads made of non-marine/terrestrial shells (i.e., ostrich eggshell) is ca. 42,000 years BP and was recovered at the neighboring site of Border Cave [55]. OES beads were also collected from late MSA/early LSA context of similar age at Apollo 11 and from layers associated with MSA industry at Boomplaas Cave [56,57]. Non-dated OES beads were also reported by Mason et al. [40] from the MSA of Cave of Hearths. Other sub-contemporaneous OES beads have been recovered north of South Africa (e.g., [58–60]).

When excavating layers 27 to 30, Eloff noticed the occurrence of hearths and provided an accurate description of artefacts distribution (seeds, scrapers, and ochre); he interpreted some of these layers as living floors. A recent publication by Badenhorst and Plug [50] offers a first description of the faunal spectrum composition, dominated by ungulate, tortoise and giant African land snail (*Achatina* sp.) remains. Of particular interest is their mention of two human remains, namely a right cuboid fragment from layer 28(a) and another cuboid fragment from layer 29(i). Unfortunately, these specimens have not been located so far and seem to have vanished, together with the whole MSA faunal collection of BRS. The presence of a few bone tools (“*a bone splinter*” in layer 28b) is suggested in Eloff’s field notes, but none of these artefacts have been located to date.

The upper MSA deposits represent finely bedded and laminated sediments that consist largely of rhombs of micritic calcite derived from the combustion of plant material. The ashes appear fresh although some are partially cemented. Some of the ashes appear laminated, suggesting that they represent primary, *in situ* combustion features (i.e., hearths). Other ashes appear to have been redeposited, most likely through anthropogenic processes, such as dumping or hearth rake-out. Carbonized and humified plant material is also present, but in varying quantities layer by layer. The reddish colour of the sediments is caused by sand-sized aggregates of silt and clay, which likely derive from soil outside of the shelter. These aggregates most likely entered the cave through slope processes. The calcareous lenses and laminations in the upper MSA deposits exhibit thin zones and crusts of phosphate, suggesting that they were exposed for a period of time, during which the ashes were altered, likely from guano. This may imply that occupations during the MSA were sporadic, allowing time for exposed surfaces to weather. No evidence for water-lain deposits has so-far been found, either in thin section or in the field.

## The luminescence chronology of the BRS Pietersburg occupations

Four sediment samples were taken from the southern profile during the 2014 field season. BRS1 and BRS2 come from the LSA sequence, while BRS3 and BRS4 belong to the upper MSA deposits (Fig 2). BRS1 was collected from layer 4 of Eloff; BRS2 and BRS3 respectively from the deposits just above (layers 17–18) and just below (layer 21) the roof debris layer that marks the separation between the LSA and the MSA; and BRS4 taken at the base of the main south profile, within layer 28(a). All samples were collected at night under subdued red-orange light.

### Equivalent doses

The samples were weighted, dried in an oven at 45°C for one week and weighed again. They were then dry-sieved for separating the >2mm from <2mm fractions. Half of the <2mm and the whole >2 mm fractions were left apart for determination of the radioisotope contents (following [61]). The second half of the <2mm fraction was then further prepared for equivalent doses (De) determination. 200–250μm (BRS1 and BRS2) or 100–140μm (BRS3 and BRS4) quartz and feldspar grains were extracted following standard procedures: wet sieving, HCl 10% (removal of carbonates), H_2_O_2_ 30% (removal of organic material), heavy liquid separation at 2.58 and 2.72 g/cm^3^ (separation of feldspars, quartz and heavy minerals). The feldspar grains were then rinsed and dried; the quartz grains were further etched for one hour with HF 40% and the feldspar grains were etched for 10 min with HF 10%. The etchings were followed by HCl 10% and a last sieving at 180 or 80 μm. The feldspar or quartz grains were spread on stainless steel discs using a 5 mm-diameter mask and fixed with silicon oil. The quartz grains were also mounted on single grain discs displaying 100 cylindrical holes 300μm deep x 300μm diameter (BRS1 and BRS2) or 150 μm x 150 **μm** (BRS3 and BRS4). Tests after [62] were performed to check for the absence of feldspar grains in the quartz extracts.

The luminescence of the single or multiple quartz grains aliquots was measured with a TL-DA/OSL 20 Risø Reader. Excitation was done either with a green laser (10 mW Nd:YVO4 emitting at 532nm) (single grains) or with blue LEDs (470 ± 20 nm) [63–65], while detection was done with a Q9235 photomultiplier tube preceded by 7.5mm of Hoya U340 filters (280–380 nm). A ^90^Sr/^90^Y beta source delivering 0.14Gy/s attached to the reader was used for irradiation. The luminescence of the feldspar grains was measured with two distinct systems: either the TL-DA/OSL 20 Risø reader, with infra-red (IR) excitation at 870 ± 30 nm and detection through Schott BG39+Corning 7–59 filters (320–460 nm), or a Smart Lexsyg system [66]. In this case, excitation was done with IR diodes (850 nm), detection with a Hamamatsu H7360-02 photomultiplier tube preceded by a combination of 3 mm Schott BG39 and 3.5 mm AHF Brightline HC filters (390–440 nm), and irradiation with a ^90^Sr/^90^Y beta source delivering 0.16 Gy/s. Analyses aimed at determining individual equivalent doses were performed with the Analyst software [67].

#### Analyses of quartz grains

**Fig 3:**
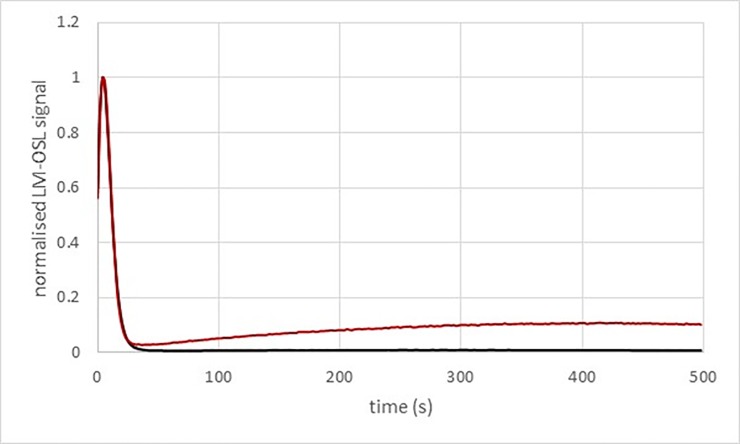
LM-OSL measurement of the BRS3 and BRS4 quartz samples (grey lines) and of the Risø calibration quartz (red lines) known to be dominated by a fast component signal. The inset shows the first 50 s of the measurements.

**Fig 4:**
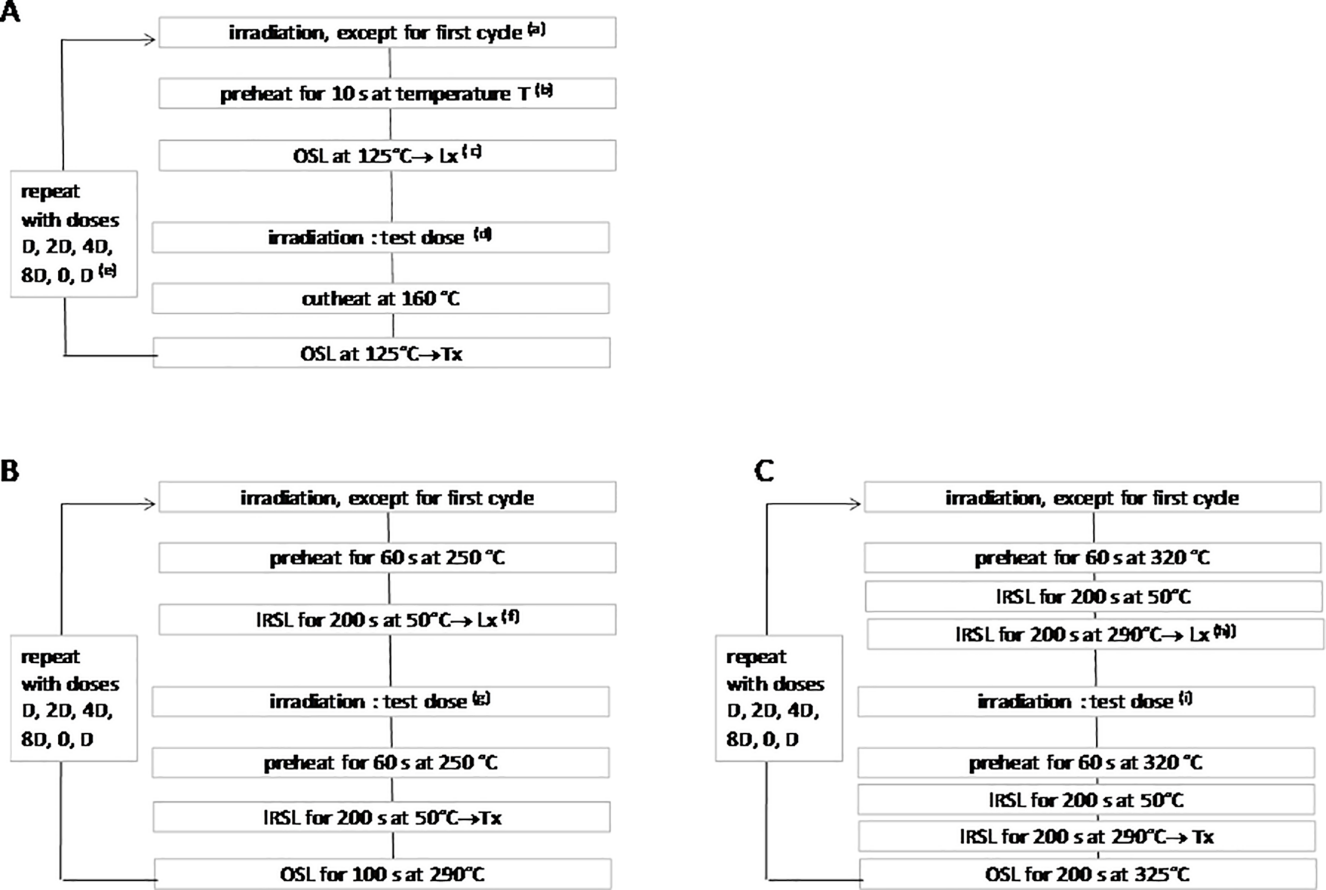
Protocols applied to the quartz or feldspar grains. A) SAR, B) IR50, C) PIR-IR290. (a) For the dose recovery tests, a dose close to the estimated De was given. (b) For the preheat plateau tests, the temperature T was set to 220, 240, 260 or 280 °C. (c) For the single quartz grains measurement, the OSL excitation was performed for 0.85 s; the first 0.05 s and last 0.1 s were taken for signal and background respectively; for the multiple quartz grains measurements, the OSL excitation was performed for 100 s; the first 2 s and last 20 s were used for signal and background respectively. (d) The test dose was either 7 Gy (BRS1 and 2) or 140 Gy (BRS3 and BRS4). (e) D was chosen close to the estimated De. (f) The first 7.6 s and last 20.4 s out of 200 s were used as signal and background respectively. (g) The test dose was either 32 Gy (BRS2) or 65 Gy (BRS3 and BRS4). (h) The first 8.0 s and last 18.4 s out of 200 s were used as signal and background respectively. (i) The test dose was either 16 Gy (BRS1 and 2) or 78 Gy (BRS3 and BRS4).

**Fig 5:**
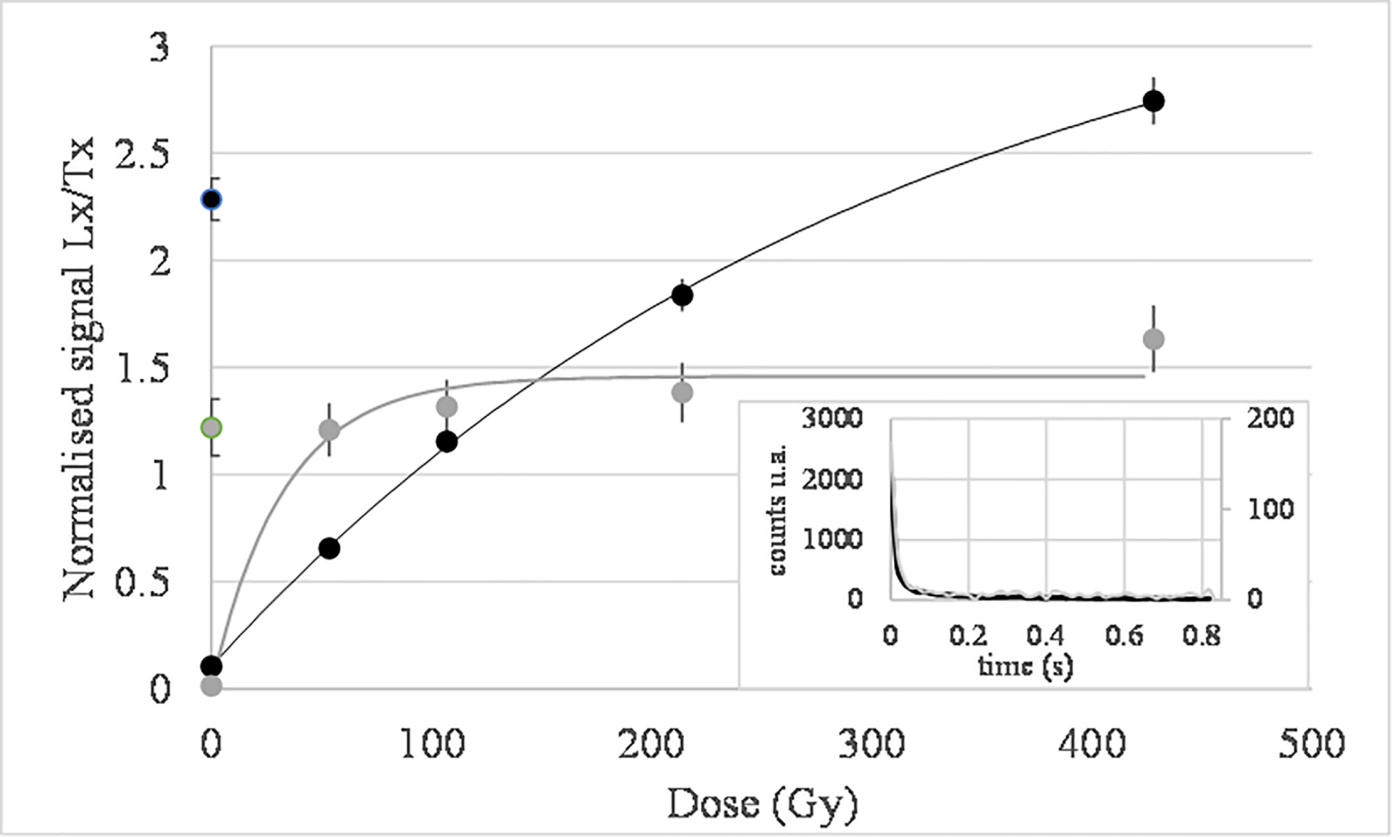
Example of two typical growth curves of BRS3 quartz grains: One (grey dots and curve) shows early saturation (D_0_ = 33 Gy) and is unable to allow measuring high De values, while the other one (black dots and curve) displays late saturation (D_0_ = 308 Gy) and can be used for measuring high De values. The inset shows corresponding natural glow curves (right axis for the black curve, left axis for the grey curve).

The luminescence signal of quartz grains from the BRS samples is bright and dominated by a fast component (Fig 3). A Single Aliquot and Regenerative dose protocol (SAR; [68,69]) was first applied to multi-grain aliquots for each quartz sample (Fig 4). A 260°C for 10s preheat was applied to the natural and regenerative doses and a 160°C cut-heat was applied to the test dose used to correct for sensitivity changes. It could be observed that the luminescence signals of the MSA samples (BRS3 and BRS4) were saturated or close to saturation. The same sequence was subsequently applied to single grains discs. It was observed that only part of the grains were actually fully saturated: it was thus still possible to work with grains whose growth curve had a large saturation level and were then able to record large De (Fig 5). Only single grain analyses were performed afterwards for quartz grains, including for the LSA samples.

Growth curves were fitted with a saturating exponential (Lx/Tx = a (1-exp((D+c)/D_0_), where D is the dose, Lx/Tx is the sensitivity corrected luminescence signal and a, c and D_0_ describe the growth curve. Grains were rejected if: 1) their signal was low (<3sigma above background), 2) the recycling ratio was not consistent with the interval [0.9; 1.1], 3) the recuperation ratio (zero dose over natural dose corrected signals) was >5%, or 4) the test dose relative error was >10%. After this selection, two groups of grains were distinguished: A) the non-saturated grains, for which a De estimate could be calculated, and B) the grains for which the Ln/Tn signal was saturated or not intercepting the growth curve. Before assessing the central De value [70], which characterizes the deposition time of each sediment sample, its dependence on the D_0_ parameter was systematically checked following [70]. Central De values were calculated (for group A), while the grains with the lowest D_0_ values were progressively discarded; in parallel the number of saturated /non intercepting grains (from group B) as a function of D_0_ was observed. The central De was estimated to be reliable when a plateau of central De values was reached and the number of rejected saturated/non intercepting grains was low (arbitrarily fixed to < 5% of the total amount of grains passing criteria i to iv, i.e., grains from A+B). This is slightly different from the criteria suggested by [71], where the threshold D_0_ value must be at least equal to the average/central De of the plateau.

**Fig 6:**
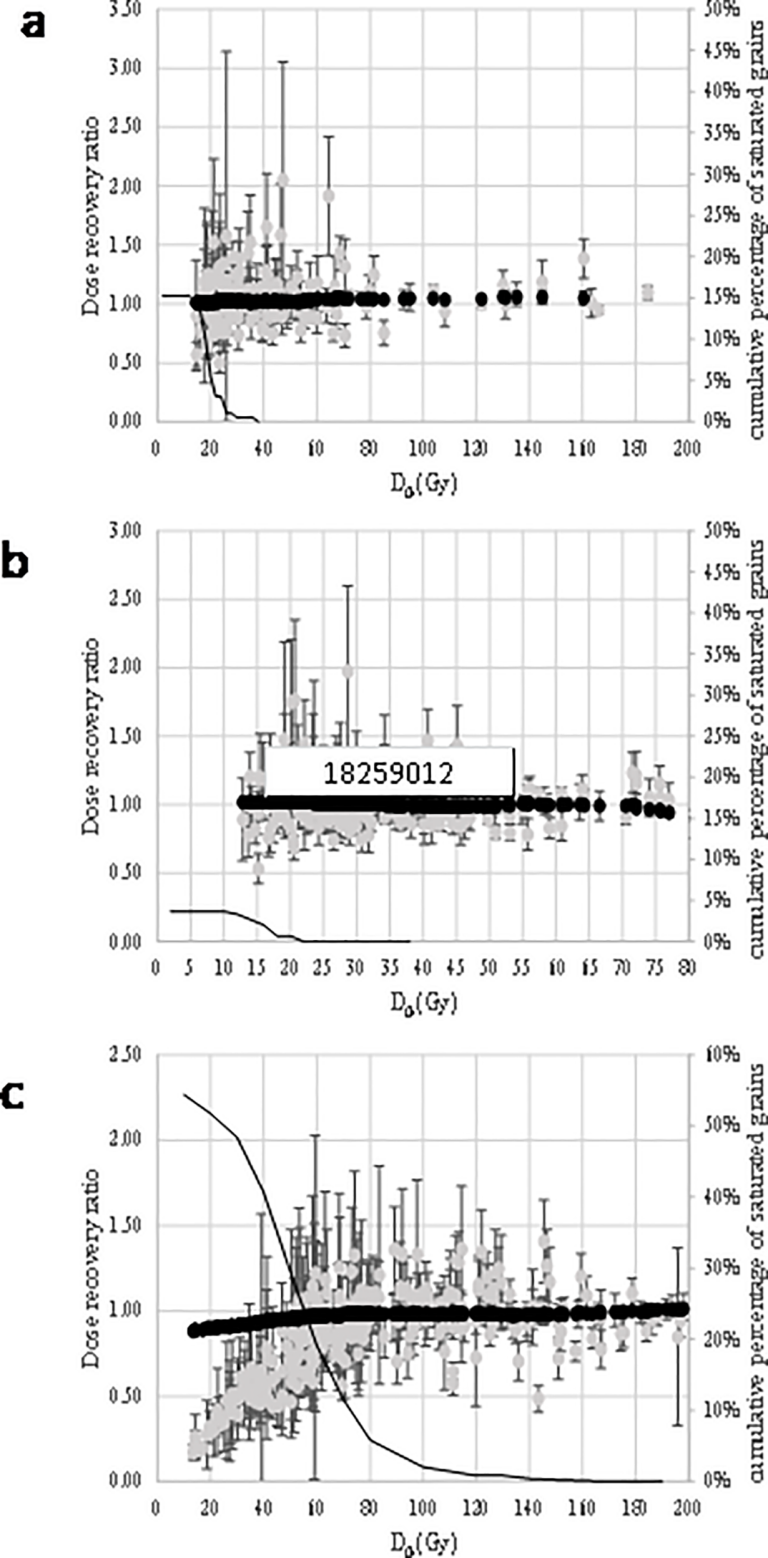
Dose recovery ratios in function of D_0_. a) BRS1, b) BRS2, c) BRS3. The grey dots represent the dose recovery ratio for each grain as a function of the D_0_ value of its growth curve. The black dots represent the central ratio while the grains with the lowest D_0_ values are progressively discard. The black line represents the cumulative number of saturated grains (in percent of the total number of grains passing criteria i to iv), as a function of the minimal D_0_ value.

Dose recovery tests were performed on BRS1, BRS2 and BRS3. No dose recovery tests were performed on BRS4 because the amount of available material was too low. The purpose of such a test is to ensure that the protocol allows the recovery of a laboratory given dose. For this test, grains were bleached for one minute in a HölneSOL500 solar simulator and were given a beta dose close to the one estimated during preliminary runs. Fig 6 displays the ratio of estimated to given dose determined for each grain as a function of D_0_; it also displays the ratio of central dose to given dose calculated while the grains with the lowest D_0_ values are progressively discarded. For BRS2, no dependence is observed and the number of saturated/non-intercepting grains is always low (<5%), so no data are further discarded. The plateau value is consistent with unity at two sigma (1.01 ± 0.01) (Fig 6B and Table 1). For BRS1, no dependence is observed even though the number of saturated/non-intercepting grains is more important (up to 15%) (Fig 6A and Table 1). For BRS3 the central De values increase with the minimal D_0_ and reach a plateau that is consistent with unity (Fig 6C). The number of rejected saturated/non intercepting grains represents up to 55% of the grains passing criteria i to iv, but decreases as expected when D_0_ increases. For a threshold value of 90 Gy (about 5% of saturated grains rejected), the dose recovery ratio is 0.98 ± 0.01. Had we strictly applied the recommendation of [71], we would have chosen a threshold value of about 167 Gy, and reached the same conclusion about the dose recovery ratio, though unnecessarily discarding most of the data.

**Table 1:** Data for the DRT estimates of the quartz grains. N: number of measured grains; saturated: number of grains that were rejected because of saturation; n1: number of grains that passed all the rejection criteria before D_0_ selection is applied; n2: number of grains that passed all the rejection criteria including D_0_ selection; D_0_: threshold for the D_0_ value; OD: over-dispersion calculated from the central age model; given dose: laboratory given dose that was expected to be recovered.

Sample	grain size	preheat	N	saturated	before D_0_ selection	after D_0_ selection	D_0_ (Gy)	OD (%)		given dose (Gy)	ratio obtained /given dose					
					n1	n2					CAM		ADM6009-55		BaSAR	
BRS1	200–250	260 10s/160°C cut	500	30	167	167	-	8	± 1	43	1.01	± 0.01	1.01	± 0.01		
BRS2	200–250	260 10s/160°C cut	500	11	288	288	-	7	± 1	30	1.02	± 0.01	1.02	± 0.01		
BRS3	100–140	260 10s/160°C cut	1200	372	305	178	90	6	± 1	161	0.98	± 0.01	0.98	± 0.01		

**Fig 7:**
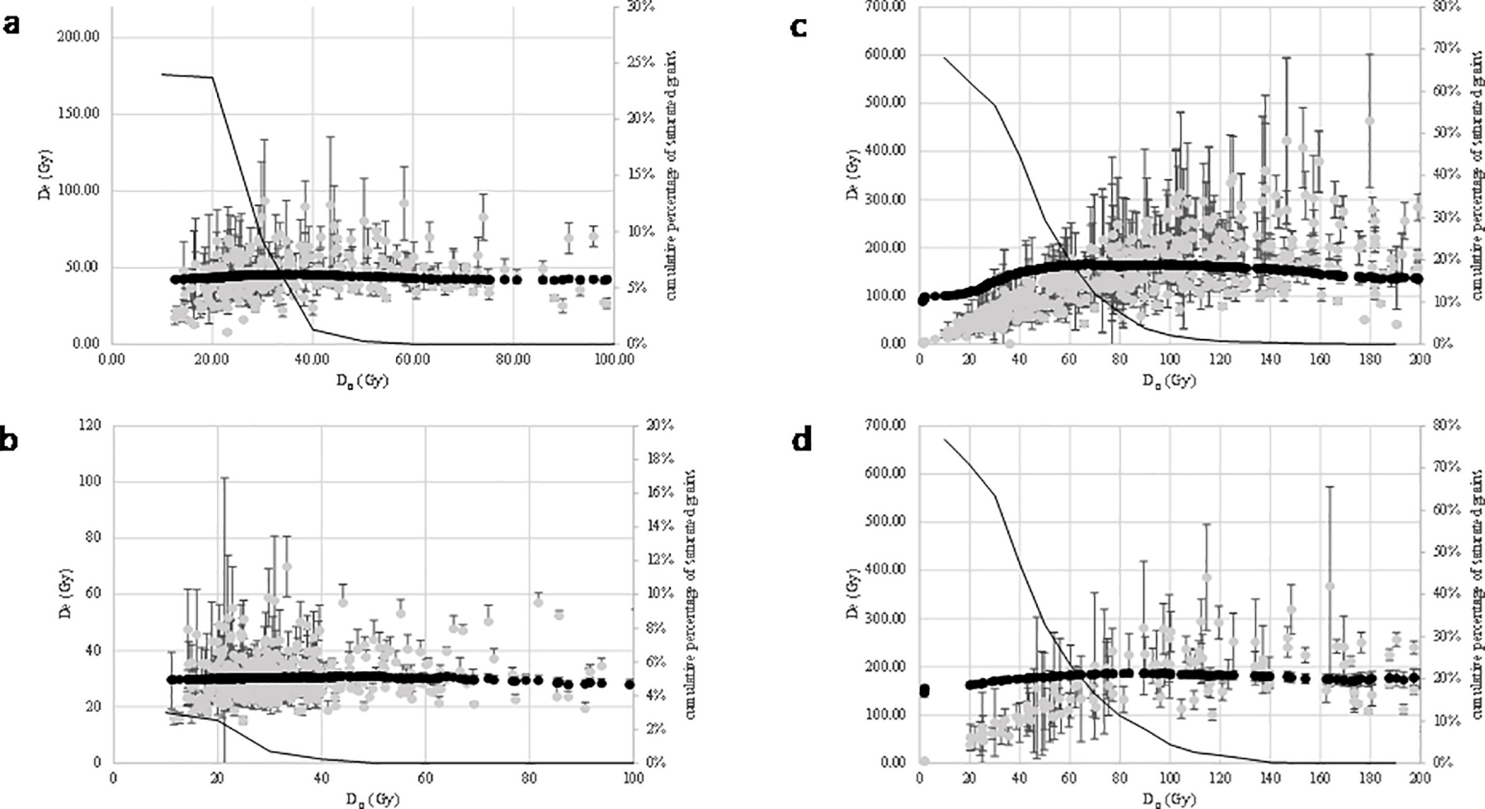
De as a function of D_0_ for each sample. The central De is calculated while the lowest D_0_ values are progressively discarded. The grey dots represent the De for each grain as a function of the D_0_ value of its growth curve. The black dots represent the central De while the grains with the lowest D_0_ values are progressively discarded. The black line represents the cumulative number of grains (in percent of the total number of grains passing the criteria) that are saturated, as a function of their minimal D_0_ value.

In addition, preheat plateau tests were performed on BRS1, BRS2 and BRS3. The purpose of this test is to ensure that the estimated De value do not strongly depend on the preheat parameters. The preheat temperature of the natural and regenerative doses was increased from 220 to 280°C by 20°C steps. For each preheat temperature, the central De and the number of saturated grains were calculated as a function of the minimal D_0_ value (Table 2). While based on the plateau and relative number of saturated/non intercepting grains, the D_0_ threshold value can vary from one preheat temperature to another, the plateau values are statistically undistinguishable for all temperatures and each sample, except may be at 280°C for BRS2. This data set has been discarded and the selected grains of the other sets have been gathered for each sample in order to calculate the final De estimates (Fig 7 and Table 2).

**Table 2:** Data for the De estimates of the quartz grains. N: number of measured grains; saturated: number of grains that were rejected because of saturation; n1: number of grains that passed all the rejection criteria before D_0_ selection is applied; n2: number of grains that passed all the rejection criteria including D_0_ selection; D_0_: threshold for the D_0_ value; OD: over-dispersion calculated from the central age model; sigma_d: dispersion calculated from the average dose model.

Sample	grain size	preheat	N	saturated	n before D_0_ selection	n after D_0_ selection	D_0_ (Gy)	OD		sigma_d		De (Gy)					
												CAM		ADM		BaSAR	
BRS1		220°C 10s-160°C cut	200	1	60	60	no	19	± 2	17	3	44.8	± 1.3	45.5	± 1.4	47.4	± 1.4
BRS1		240°C 10s-160°C cut	200	10	51	20	40	13	± 3	10	4	48.1	± 1.8	48.3	± 1.9	50.0	± 1.8
BRS1		260°C 10s-160°C cut	500	36	155	62	40	33	± 2	21	4	43.7	± 1.4	44.7	± 1.4	45.7	± 1.5
BRS1		280°C 10s-160°C cut	200	47	33	20	40	21	± 4	21	4	50.2	± 2.9	51.3	± 3.4	51.9	± 3.8
**BRS1**	**200–250**	**220–280**	**1100**	**94**	**266**	**142**	** **	**21**	**± 2**	**19**	**2**	**45.4**	**± 0.9**	**46.2**	**± 0.9**	**47.6**	**± 0.9**
BRS2		220°C 10s-160°C cut	200	2	92	92	no	18	± 2	17	3	31.2	± 0.7	31.6	± 0.7	32.3	± 0.7
BRS2		240°C 10s-160°C cut	200	1	109	109	no	19	± 2	17	2	28.9	± 0.6	29.4	± 0.7	29.3	± 0.5
BRS2		260°C 10s-160°C cut	500	10	221	189	20	19	± 1	18	2	29.9	± 0.5	30.3	± 0.5	30.6	± 0.5
BRS2		280°C 10s-160°C cut	200	20	40	40	no	27	± 4	26	5	41.3	± 2.0	42.7	± 2.8	44.4	± 1.7
**BRS2**	**200–250**	**220–260**	**900**	**13**	**422**	**390**	** **	**19**	**± 1**	**18**	**1**	**29.9**	**± 0.3**	**30.4**	**± 0.4**	**30.5**	**± 0.3**
BRS3		220°C 10s-160°C cut	1100	479	180	104	100	40	± 3	37	5	165.5	± 7.2	177.5	± 6.2	192.3	± 7.3
BRS3		240°C 10s-160°C cut	1100	485	173	108	80	27	± 2	22	3	153.9	± 4.5	157.8	± 4.5	170.6	± 5.2
BRS3		260°C 10s-160°C cut	1100	485	181	90	90	29	± 3	25	3	171.3	± 5.9	176.8	± 5.9	180.8	± 7.5
BRS3		280°C 10s-160°C cut	1000	300	207	81	80	33	± 3	29	4	149.2	± 6.1	155.8	± 6.8	162.1	± 6.7
**BRS3**	**100–140**	**220–280**	**4300**	**1749**	**741**	**383**	** **	**33**	**± 1**	**30**	**2**	**160.0**	**± 3.2**	**167.2**	**± 3.1**	**176.7**	**± 3.3**
**BRS4**	**100–140**	**260°C 10s-160°C cut**	**1600**	**459**	**139**	**66**	**110**	**35**	**± 4**	**28**	**4**	**181.1**	**± 7.9**	**191.4**	**± 8.4**	**192.3**	**± 8.7**

While applying the Central Age (actually dose) Model (CAM; [70]), it is assumed that the distribution is consistent with a single well bleached, undisturbed population of grains. This assumption is sustained at least for BRS1 and BRS2 thanks to the comparison with feldspar and radiocarbon age estimates (see below). However, recent criticisms [72,73] have suggested that the CAM could lead to age underestimates, in particular for old samples. Firstly, it was noticed that the error estimates are not properly treated, because of their propagation as Gaussian error estimates while they are not. The Bayesian SAR model (baSAR) proposed by [74] has been developed to overcome this drawback. Secondly, it was noticed that the CAM De is a geometric mean, while the dose rate (Dr) is an arithmetic mean; therefore, dividing the CAM De by the Dr is not correct. While this statement has been contested by Galbraith [75], Guérin et al. [73] proposed the Average Dose Model (ADM), an alternative to the CAM to correct for this eventual bias. The debate on the accuracy and relevance of the different equivalent dose estimate is still open and no consensus has yet been found. Therefore, in addition to the CAM De, both the ADM and baSAR De estimates have been calculated (Table 2).

It was first checked that the dose recovery ratios were still consistent with unity using these statistical models (Table 1). For the De data sets, the ADM De values are always higher than the CAM De values, though consistent either at one (BRS1, BRS2 and BRS4) or two sigma (BRS3). This can be related to the fact that the De distributions of the selected grains are not strongly positively skewed, and the over-dispersion is relatively low (< 35 ± 4%), limiting in this case the drawbacks of the CAM. The baSAR De values are consistent at one sigma with the ADM values (BRS1, BRS2 and BRS4) or slightly higher (BRS3), though within two sigma, as expected as a consequence of different error treatments.

#### Analyses of the feldspar grains

Two single aliquots (multi-grain) protocols were applied for the De estimate of the feldspar grains: the InfraRed Stimulated Luminescence (IRSL) at 50°C protocol (IR50) after Auclair et al. [76] and the Post IR-IRSL at 290°C protocol (PIR-IR290) after Thomsen et al. [77] and Buylaert et al. [78] (Fig 4B and 4C). There were not enough feldspar grains for BRS1 and, thus, only the PIR-IR290 was applied to this sample. As for the quartz estimates, the recycling ratio, recuperation and signal intensities were looked at for both protocols. Contrary to quartz, no saturated signals were observed. For the IR50 protocol, fading rates were measured (Table 3) and corrections were applied following the Dose rate Correction (DRC) model of Lamothe et al. [79]. For the PIR-IR290 protocol, no correction was made either for the residual dose or fading, following Thomsen et al. [80]. Simple arithmetic means were applied to calculate the mean De of each distribution (Table 3) (other more complex models would be of no interest here since the individual error estimates of multi-grain aliquots are not significantly different). The De values obtained with the two protocols are consistent at one sigma (BRS3 and BRS4) or two sigma (BRS2).

**Table 3:** Data for the De estimates of the feldspar grains. n: number of aliquots; g: mean g value n % per decades for the fading correction; corrected De (Gy): fading corrected De value. The DRC method of [79] was applied.

Sample	grain size	PIR-IR290			IR50				
		n/N	De (Gy)		n/N	g		corrected De (Gy)	
BRS1	200–250	5/8	46.7	± 2.7				nd	
BRS2	200–250	8/11	45.3	± 3.5	12/12	2.4	0.3	38.0	± 1.7
BRS3	100–140	14/14	261.2	± 23.2	12/12	3.7	0.6	285.5	± 25.3
BRS4	100–140	16/16	308.3	± 23.2	11/12	3.3	0.4	289.6	± 19.7

### Dose rates

Four contributions were considered to estimate the Dose rate (Dr): the cosmic, gamma, beta and alpha dose rates. For the beta and alpha dose rates, we also distinguished the contribution from inside the grains from that of their environment. Water content attenuates the radiations and the current water content was thus measured. Field observations indicate that ashes are very well preserved in the archaeological sequence, and thin sections show that there is no evidence for any significant diagenesis. These geoarchaeological observations suggest very limited water percolation, if any. Therefore, one can assume that the present low water content is representative of the past mean water content (Table 4). This would also imply no significant change in the radioisotope content (but for eventual radon escape), and then a constant dose rate over time which is a basic requirement for luminescence dating.

**Table 4:** Data for the De estimates. Coarse fraction content: weight fraction of the [2mm-1 cm] material over the total (< 1 cm) sediment. Cosmic dose rate are calculated from [80]; gamma dose rates are deduced from in situ dosimetry [81,82]; beta 1 and beta2 are calculated from the activities measured with high resolution gamma spectrometry (see Table 5) using specific dose rates of [83] and attenuation factors of [83]. Beta1 is calculated from the activities of the fine (< 2 mm) fraction; beta2 is calculated from the activities of the total (< 1 cm) fraction. Internal dose rate of quartz was neglected; internal dose rate of feldspar is deduced from the measured K content and from an assumed 400 ppm of Rb, following [84]. Total dose rates take into account the mean of beta1 and beta2 (according to [61,85]). Uncertainties included both systematic and statistic errors.

Sample	grain size	water content	coarse fraction content	Dose rates (Gy/ka)													
				cosmic		gamma		beta1		beta 2		internal (feldspaths)		total quartz		total feldspaths	
BRS1	200–250	3%	18%	0.06	± 0.03	1.52	± 0.09	1.99	± 0.21	2.16	± 0.22	1.00	± 0.04	**3.65**	**± 0.24**	**4.65**	**± 0.24**
BRS2	200–250	3%	35%	0.06	± 0.03	0.83	± 0.04	1.32	± 0.14	1.14	± 0.12	1.00	± 0.04	**2.12**	**± 0.14**	**3.12**	**± 0.15**
BRS3	100–140	3%	33%	0.06	± 0.03	0.67	± 0.03	1.76	± 0.17	1.36	± 0.14	0.60	± 0.03	**2.29**	**± 0.16**	**2.88**	**± 0.17**
BRS4	100–140	4%	14%	0.06	± 0.03	0.80	± 0.04	1.70	± 0.17	1.72	± 0.18	0.60	± 0.03	**2.57**	**± 0.18**	**3.16**	**± 0.19**

The cosmic Dr was calculated after the equation of Prescott and Hutton [86], using an estimate of the roof thickness above the excavation (10m) and taking the geometry of the cave into account (Table 4). The gamma Dr was estimated using Al_2_O_3_:C dosimeters, left within the sections for 9 to 12 months at the location where the sediment samples had been taken [87]. The gamma dose rate stands between 0.67 and 0.83Gy/ka for the dosimeters associated with BRS2, BRS3 and BRS4, but is much larger for the dosimeter associated with BRS1 (1.52 Gy/ka). This is likely due to the high K content of the layer where this dosimeter was inserted, as suggested by the internal radio-isotopic content of BRS1 (Table 5).

**Table 5:** Activities of the ^238^ U, ^232^ Th decay chains and for ^40^K and calculated beta dose rates. For the Uranium family, the activities deduced from the ^234^Th (pre ^226^Ra–“top”), from the ^214^Bi and ^214^Pb contents (post ^226^Ra_ “middle”), and from ^210^Pb (“bottom”) are displayed. a) fraction< 2 mm, b) fraction > 2 mm and < 1 cm, c) mean activity and dose rate calculated from a) and b), taking into account the relative weight of each fraction.

	Sample BRS	Activities (Bq/kg)								Content (%)	
		^238^ U series						^232^Th series		K	
		Top		Middle		Bottom					
fraction < 2 mm	1	19.0	± 2.5	20.8	± 0.6	20.0	± 2.7	32.0	± 3.2	2.34	± 0.03
	2	14.5	± 1.7	12.5	± 0.3	11.5	± 2.8	17.2	± 1.5	1.58	± 0.03
	3	23.2	± 3.0	17.5	± 0.4	15.9	± 2.6	32.8	± 3.3	1.99	± 0.03
	4	17.5	± 2.2	13.5	± 0.3	13.1	± 2.4	27.0	± 2.5	1.89	± 0.03
fraction > 2mm	1	33.0	± 3.7	27.3	± 0.7	26.9	± 2.2	46.3	± 4.9	3.47	± 0.04
	2	6.6	± 0.3	4.9	± 0.1	9.6	± 1.1	4.7	± 0.1	1.06	± 0.02
	3	6.4	± 0.3	3.9	± 0.0	4.9	± 1.0	5.8	± 0.2	0.63	± 0.01
	4	14.4	± 1.0	10.8	± 0.2	11.7	± 1.4	20.3	± 1.2	1.56	± 0.02
total	1	21.5	± 2.7	21.9	± 0.6	21.2	± 2.6	34.5	± 3.6	2.54	± 0.03
	2	11.7	± 1.4	9.8	± 0.3	10.8	± 2.3	12.8	± 1.2	1.40	± 0.02
	3	13.4	± 1.9	9.6	± 0.3	9.5	± 1.8	17.0	± 2.1	1.20	± 0.02
	4	16.0	± 1.7	12.2	± 0.3	12.5	± 2.0	23.8	± 2.0	1.73	± 0.02

We assumed that the HF etching had fully removed the outer layer of the grains irradiated by the external alpha rays. The external beta Dr was estimated from high resolution gamma spectrometry (Table 4). As suggested by Martin [85] and Tribolo et al. [61], an attempt was made to improve the beta Dr estimate by taking into account the difference of radio-isotopic contents and auto-absorption depending on the size of the material. For this reason, the <2 mm and >2 mm fractions were analyzed separately. For BRS3 and BRS4, that presented a significant amount of material > 1 cm, this one was discarded. Indeed, the auto-absorption of the > 1 cm material is so high that its contribution to the irradiation of the sedimentary quartz and feldspar grains can be neglected [88]. The preparation of the sample for these measurements required fine crushing of the different fractions and sealing in plastic boxes. Measurements were performed after at least one month, in order for the post ^226^Ra radioisotopes to achieve equilibrium with their parents. Table 5 presents the activities for the different fractions and for the whole sample (excluding > 1cm material), taking into account the relative weight of the > 2 mm and < 2 mm fractions. For BRS2 and BRS3, the activities or contents of the coarse fraction are significantly lower than the activities/contents of the fine fraction, while for BRS1, they are significantly higher. The activities/contents for BRS4 > 2 mm or <2 mm are not significantly different. Dose rates were calculated using attenuation factors of Guérin et al. [89] and specific dose rates of Guérin et al. [83]. Tribolo et al. [61] (see also [85]) have argued that the true beta dose rate stands between the dose rates estimated from the fine and the fine+coarse fractions. Indeed, in case the internal dose rate in the coarser fraction is higher than in the fine fraction, the quartz grains near the coarse material will experience a higher dose rate than those, which are further apart. The true mean dose rate for all the quartz grains is thus higher than the dose rate considering only the content of the fine material. On the other hand, when calculating the dose rate from the meanfine+coarse content, we do not take into account the fact that the contribution from the coarse material in actually tempered by some auto-absorption, i.e. the dose rate is overestimated. A similar reasoning can be applied when the content of the coarse fraction is lower than in the fine fraction. The dose rates from the fine and fine+coarse fractions are up to 23% (BRS3) different. The mean between “fine” and “fine+coarse” dose rates were used for the final age estimates (Table 4).

For the quartz grains, the internal Dr was assumed to be negligible. For the feldspar grains the internal Rb content was assumed to be 400 ppm, as suggested by Huntley and Hancock [84]. For the K content, SEM-EDX measurements were first attempted on individual grains, for which luminescence signal intensity had been previously checked. Few grains seemed to have an actual K content close to the published value (12.5%) of Huntley and Baril [90]. However, the measurements were likely biased by remains of silicon oil that were used during the preliminary luminescence measurements. A value of 12.5±2% has thus been used for the calculation but might be seen as a maximum estimate. The HF etching was taken into account for the calculation of the internal dose rate, which is thus 1.00 ± 0.04 Gy/ka for 200–250 μm feldspar grains and 0.60 ± 0.03 Gy/ka for 100–140 μm feldspar grains (Table 4).

The total dose rates stand between 2.12 ± 0.14 and 3.65 ± 0.24 Gy/ ka for the quartz grains and 2.88 ± 0.17 and 4.65 ± 0.24 Gy/ka for the feldspar grains (Table 4).

### Ages

**Fig 8:**
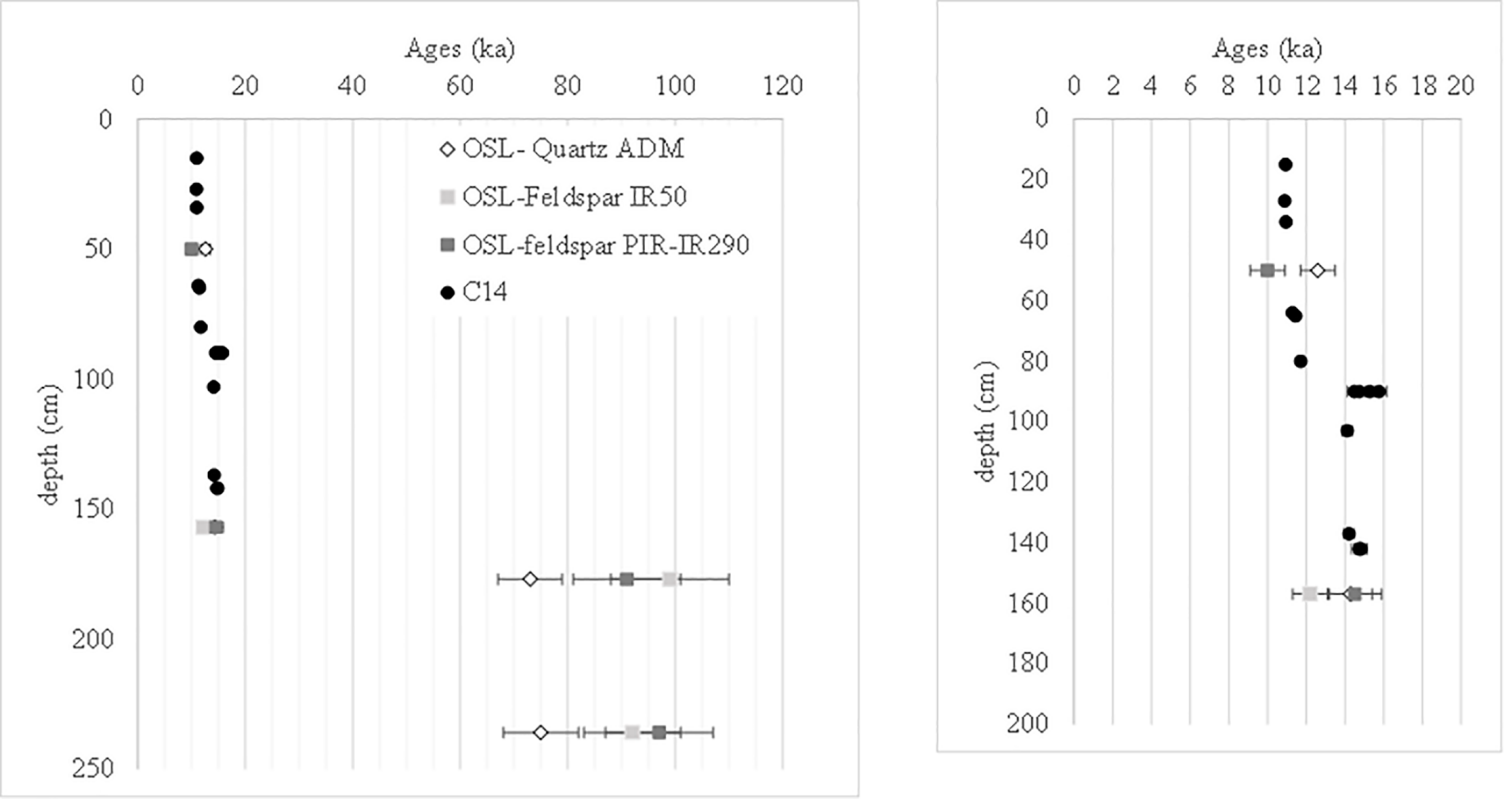
Ages in function of the relative stratigraphic location. The inset displays a zoom on the LSA sequence.

Ages for BRS1 (Eloff’s layer 5/6), BRS2 (layer 18), BRS3 (layer 21) and BRS4 (layer 28a) are presented in Table 6 and Fig 8. For BRS2, BRS3 and BRS4, the PIR-IR290 and IR50 age estimates are consistent with one another. Moreover, the PIR-IR290 age for BRS2 (14.5 ± 1.4 ka) is consistent with the radiocarbon age estimates (about 15.5 ka cal BP). This would suggest that this sample has been properly bleached before deposition and that fading issues have been properly handled. However, the PIR-IR290 age estimate for BRS1 (10.0 ± 0.9 ka) and IR-50 age estimate for BRS2 (12.2 ± 0.9 ka) slightly underestimate the expected radiocarbon ages (11.5 and 15.5 ka cal BP respectively); the feldspar age estimates for BRS3 and BRS4 (mean 91± 10 ka and 97± 10 ka respectively) are significantly higher than the quartz age estimates (70± 6 to 77± 6ka for BRS3 depending on the De model and 71± 6 to 75 ± 7ka for BRS4). The reasons for these discrepancies are unclear: on the one hand, overestimation of the internal K content could induce an underestimate of the true age (BRS1 and BRS2?). On the other hand, poor bleaching would imply overestimate of the true age (BRS3 and BRS4?), although we might then expect the PIR-290 age to be systematically significantly higher than the IR50 age estimate, which is not the case.

**Table 6:** Ages. Three estimates are given using data from Tables X2b, X3 and X4: either the ADM model for single quartz grains or the PIT-IR290 or IR50 ages for feldspar grains. A 4% systematic uncertainty has been included quadratically for taking into account the uncertainty of the calibration of the radioactive sources.

Sample	Depth (cm)	Layer (Eloff)	Cultural attribution	Age (ka)									
				CAM quartz		ADM quartz		ADM quartz		IR50 feldspaths		PIR-IR290 feldspaths	
BRS1	50	5/6	LSA	12.4	± 0.9	12.6	± 0.9	12.6	± 0.9	nd	nd	10.0	± 0.9
BRS2	157	18		14.1	± 1.0	14.3	± 1.1	14.3	± 1.1	12.2	0.9	14.5	± 1.4
BRS3	177	21	MSA	70	± 6	73	± 6	73	± 6	99	± 11	91	± 10
BRS4	236	28a		71	± 6	75	± 7	75	± 7	92	± 9	97	± 10

Meanwhile, both the quartz and feldspar ages show that the LSA and MSA layers are separated by a significant chronological and sedimentological gap, spanning at least 46 000 years. The ages also indicate that the MSA sequence predates the MIS4. Further analyses are in progress for refining the time frame, namely applying TL dating to burnt lithics and U-series on shells.

## Lithic technology of the ‘28’ Pietersburg at Bushman Rock Shelter

### Methodology and analysed sample

Here we aim at providing a general introduction to the MSA collection recovered during our 2016 excavation, which includes the SUs Gig to Gotlib. The 2016 lithic collection comprises 1441 plotted lithic artifacts, to which 145 lithic pieces larger than 20 mm extracted from the sieving could be added. Significant discrepancies are observed between the SUs in terms of lithic density, with a variation from 1 to 10 pieces (>20 mm) per liter of sediment.

The lithic assemblage is dominated by two types of raw material, namely hornfels and quartz. We acknowledge difficulties in discriminating hornfels from finer varieties of dolerite and chert and refer to the classification of Plug [53], who equates hornfels with lydianite. In the absence of petrographic characterization and in order to relate more closely the classification with our technological study, we subdivided the hornfels into three categories that correspond to fine, medium and coarse hornfels. This classification, based on naked eye observations, is subjective and based on the roughness of the fracture surfaces and the presence of crystals. Other raw materials include quartzite and chert. Our preliminary survey of the primary and secondary geological formations surrounding the shelter suggests that all the rocks may have a local origin.

Regarding the techno-typological characterization, we followed two main steps. The first analytical step was to assess the techno-economical variability within the phase ‘28’, in terms of raw materials, blanks and formal tool distribution. The second step follows the general principles of the *chaîne opératoire* approach [91–95], which aims at recognizing the different stages of the reduction sequence and the set of geometric rules configured by the knappers. This step integrates a preliminary description of the typological corpus. The following description is based on our 2016 record but incorporates some observations based on selected specimens of layer 28 from Eloff’s collection as well (those pieces, however, are not included in counts and other quantitative analyses).

### Results: Presentation of the lithic assemblage

The lithic assemblage (n = 1586) is dominated by hornfels, which composes 50% to 90% of the collection (Table 7), but also involves a fair amount of quartz that ranges from ca. 5% to 40%. Other raw materials are represented by a handful of specimens made of quartzite and other fine siliceous rocks.

**Table 7:** Raw materials per Stratigraphic Unit (N = total number per SU).

	Hornfels Fine	Hornfels Medium	Hornfels Coarse	Hornfels (Total %)	Quartz	Quartzite	Others	TOTAL
**GIG (N = 47)**	30	28	13	*(71)*	6	17	6	100%
**GINA (N = 75)**	30	50	8	*(88)*	4	6.5	1.5	100%
**GINIUS (N = 22)**	40	37	0	*(77)*	9.5	13.5	0	100%
**GIPSY (N = 132)**	29	29.5	8.5	*(57)*	16	12	5	100%
**GIRO (N = 218)**	32.5	19	12	*(63*.*5)*	26	10	0.5	100%
**GISEL (N = 298)**	19	30	19.5	*(58*.*5)*	23.5	5.5	2.5	100%
**GIVEN (N = 301)**	29.5	29	15.5	*(74)*	24.5	0.5	1	100%
**GIX (N = 52)**	28.5	20	4	*(52*.*5)*	42	5.5	0	100%
**GO (N = 62)**	3.5	21	27.5	*(52)*	45	0	3	100%
**GODOT (N = 74)**	11.5	23	16	*(50*.*5)*	33.5	9.5	6.5	100%
**GONZO (N = 120)**	21	31	16	*(68)*	25.5	2.5	4	100%
**GOTLIB (N = 185)**	22.5	19.5	11	*(54)*	41.5	2.5	3	100%

Cortex identified on some lithic pieces indicates that hornfels and quartz were collected in different places. Cortical flakes made on hornfels dominantly show weathered natural surfaces characteristic of collection in sub-primary outcrops, while cortical pieces made on quartz have a rolled cortex that reflects collection in alluvial formations. A secondary context of collection is also inferred from quartzite and chert.

There is a paucity of cores in our lithic collection, only represented by nine specimens of which five are on quartz and two on quartzite. However, our technological classification (Table 8) displays a fair proportion of cortical flakes, small flakes and fragments <20 mm, which implies that some knapping events occurred *in situ*. We suggest our lithic assemblage reflects different techno-economical dynamics with products such as cores, blanks and formal tools that were transported into, within and away from the shelter. Laminar products dominate the lithic assemblage (one exception being the SU Go) with proportions ranging from 30% to 75% of the collection (Table 8). Amongst the set of small flakes, it is worth mentioning the presence of characteristic retouch flakes (see [96] for a definition of attributes), indicating that some of the tools were shaped and sharpened *in situ*. With regard to the typological corpus, the total assemblage consists of three main categories: end-scrapers, side-scrapers and notches/denticulates (Table 9). The proportion of formal tools varies from 0% to 7.5% of the assemblages.

**Table 8:** Technological classification per Stratigraphic Unit (N = total number per SU).

	Cortical (≥25%) flakes	Flakes	Bipolar flakes	Triangular Flakes	Laminar Flakes	Blades	Bladelets	Cores	Manuports	Fragments ≥20mm	TOTAL ≥20mm	Lithics <20mm	TOTAL (N)
**GIG**	7	16	0	0	6	11	7	0	0	0	**47**	354	**448**
**GINA**	6	28	0	0	15	11	15	0	0	0	**75**	536	**686**
**GINIUS**	0	2	3	0	1	6	10	0	0	0	**22**	214	**258**
**GIPSY**	7	53	1	0	15	28	26	0	0	2	**132**	944	**1208**
**GIRO**	10	78	2	1	26	50	48	0	0	3	**218**	1705	**2141**
**GISEL**	18	139	2	2	30	60	41	3	3	0	**298**	1603	**2199**
**GIVEN**	10	131	2	0	17	40	93	2	2	4	**301**	1272	**1874**
**GIX**	1	23	0	0	1	11	16	0	0	0	**52**	377	**481**
**GO**	4	25	10	0	5	2	1	2	0	13	**62**	1346	**1470**
**GODOT**	12	29	5	3	14	9	0	1	0	1	**74**	1337	**1485**
**GONZO**	13	43	9	4	16	17	1	0	0	17	**120**	1014	**1254**
**GOTLIB**	15	45	7	1	25	37	30	1	0	24	**185**	1171	**1541**
**TOTAL (N)**	**103**	**612**	**41**	**11**	**171**	**282**	**288**	**9**	**5**	**64**	**1586**	**11873**	**15045**

**Table 9:** Typological classification per Stratigraphic Unit (N = total number per SU).

	End-scrapers	Side-scrapers	Convergent scrapers	Denticulates	Notches	Others	TOTAL	% of Tools
**GIG (N = 47)**	1	2	0	0	0	1	**4**	8.5%
**GINA (N = 75)**	2	0	0	0	1	0	**3**	4%
**GINIUS (N = 22)**	1	0	0	0	0	0	**1**	4.5%
**GIPSY (N = 132)**	0	0	0	0	0	0	**0**	0
**GIRO (N = 218)**	8	0	1	0	2	1	**12**	5.5%
**GISEL (N = 298)**	9	2	0	0	0	4	**15**	.5%
**GIVEN (N = 301)**	7	7	0	1	0	0	**15**	5%
**GIX (N = 52)**	0	0	0	0	0	0	**0**	0
**GO (N = 62)**	3	0	0	0	0	1	**4**	6.4%
**GODOT (N = 74)**	4	0	0	3	1	0	**8**	10.8%
**GONZO (N = 120)**	1	3	1	0	5	0	**10**	8.3.%
**GOTLIB (N = 185)**	2	4	1	1	1	0	**9**	4.9%
**TOTAL (N = 1586)**	**39**	**18**	**3**	**5**	**10**	**7**	**82**	5.2%

There is substantial variation between the SUs with regard to their petrographic, technological and typological composition. Further studies will have to address the significance of this observation. However, at this stage of our analysis, we are inclined to consider the 2016 collection as one assemblage being characterized by the dominance of hornfels (66% of the whole assemblage), the production of laminar blanks (47% of the whole assemblage) and the manufacture of end- and side-scrapers. We propose that quantitative variations observed relate -not exclusively- to the low sample size of each SU, to differences in spatial occupations of the site and/or to changes in site function.

### Techno-typological characteristics

The 2016 lithic assemblage documents reduction sequences orientated towards laminar flakes (Length Index (LI)>1.5), blades (LI>2) and bladelets (LI>2 and width ≤11mm). These laminar products respectively compose 11%, 18% and 18% of the lithic assemblage (Table 8).

The laminar collection is largely composed of fragmented pieces as we only count a total of 30% of complete laminar flakes, 10% of complete blades and 16% of complete bladelets. These fractures may have occurred during use or after deposition, but a large proportion of the fractures (see [92]) seems to have originated at the time of blank production (the fracture occurring in relation to the rock properties -its elasticity and homogeneity-, the configuration of the cores and the nature of the techniques used).

**Fig 9:**
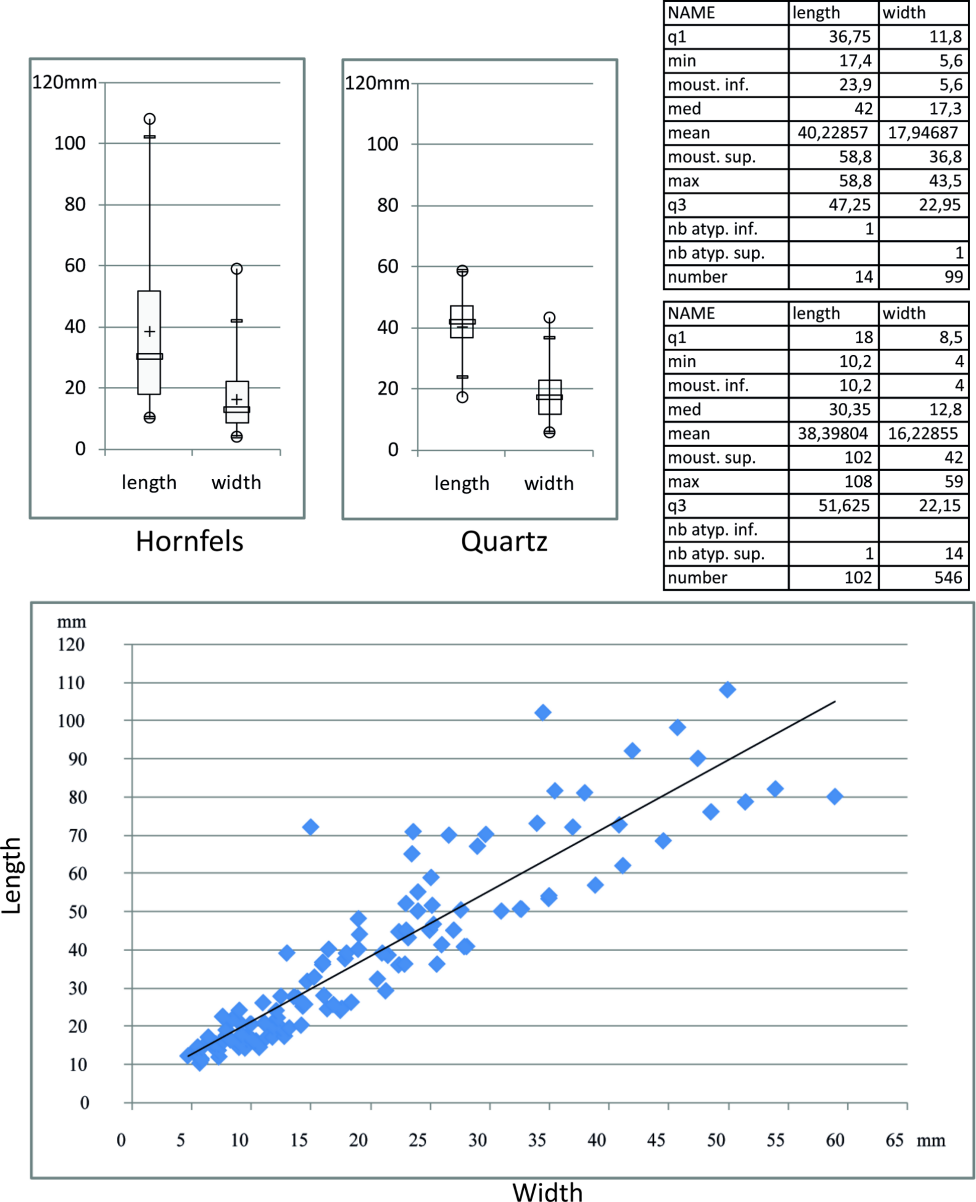
Dimensional variability of the blades from the Pietersburg phase ‘28’ of Bushman Rock Shelter.

The laminar sample is characterized by a mean length of 38.7 mm (from a maximum of 106 mm to a minimum of 10 mm), a mean width of 16.8 mm (from a maximum of 59 mm to a minimum of 4 mm) and a mean thickness of 4 mm (from a maximum of 16 mm to a minimum of 0.5 mm) (Fig 9). The data indicate that a wide range of dimensional products were obtained but also highlight a blade production that was orientated towards large specimens (mean width of 22 mm).

Laminar blanks vary in terms of morphology. Nevertheless, preliminary observations, to be supported by quantitative data, allow us to postulate the dominance of (sub)parallel forms over convergent ones, and of trapezoidal sections over triangular ones (either symmetric or asymmetric), as well as the dominance of straight profiles (with only a few twisted examples).

**Fig 10:**
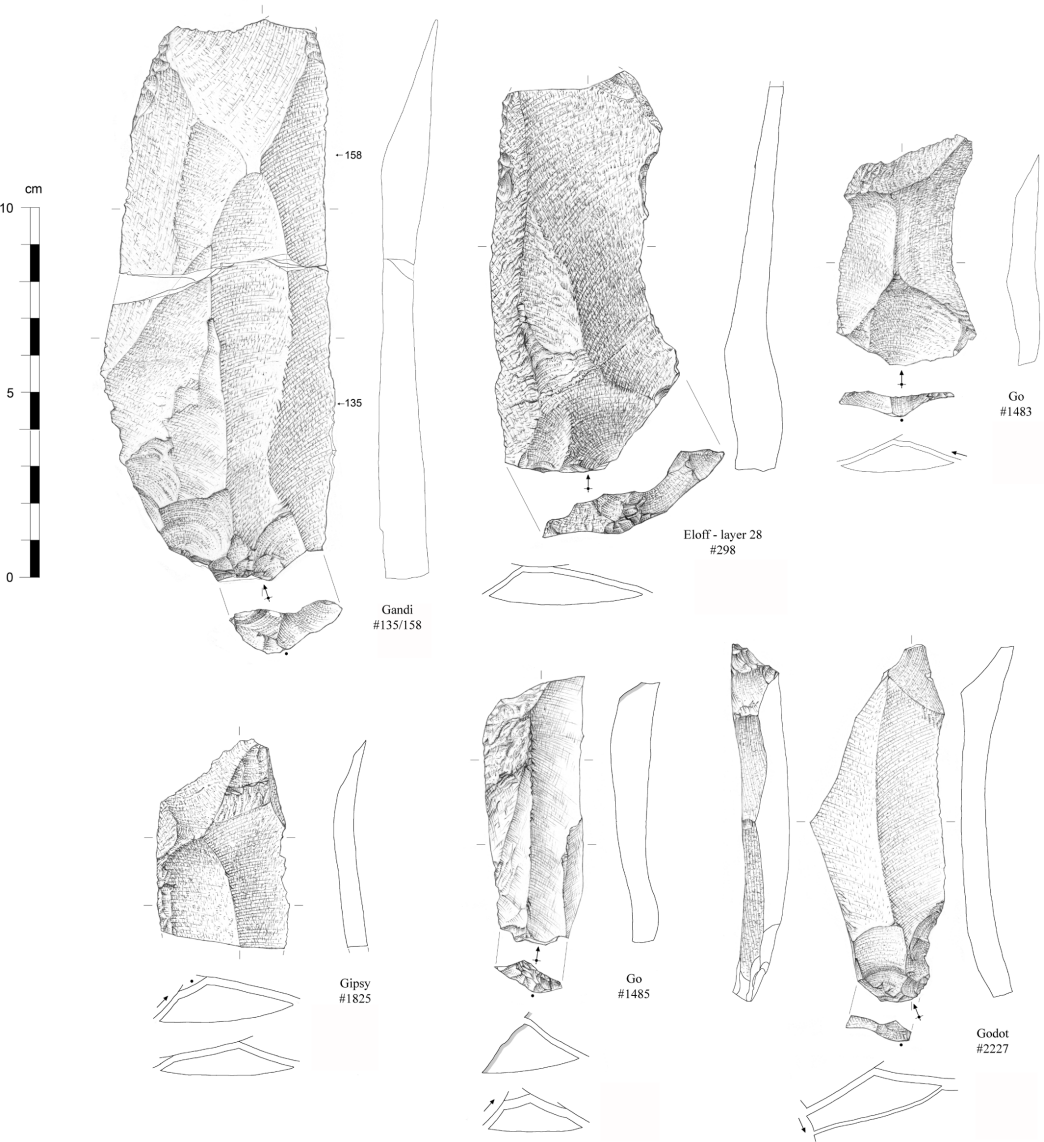
Blanks from the Pietersburg phase ‘28’ of Bushman Rock Shelter (#135/158, #298 and #1825: Levallois blades; #1483: Levallois flake; #1485 and #2227: “Prismatic” blades) (drawings by M. Grenet).

Three of the cores can be classified as laminar. Together with the description of the blanks and on the basis of mental refitting [91,97], we are inclined to recognize two distinct laminar reduction sequences. The first and main reduction method can be classified as Levallois [98,99]. One core in quartz fits such criteria: it shows one main surface of exploitation with unidirectional removals, an orthogonal preparation and a prepared platform with angles comprised between 80° and 90°. Typical Levallois blades and laminar flakes are present in the collection (Fig 10); they document a dominant unidirectional reduction with orthogonal to centripetal preparation of the surface.

The second reduction sequence is consistent with a semi-prismatic exploitation of the core volume. One core in quartzite and a second one in chert fall into this category though their stages of discard do not allow a formal description of their geometric configuration. The blanks that are assigned to this reduction strategy (blades with a “prismatic” section, Fig 10) indicate a dominant unidirectional production. The presence of a few plunging blades and cortical blades, and the absence of crested blades suggest little preparation of the surface prior to exploitation. One blade with a Kombewa residual surface on its distal end suggests the knappers may have taken advantage of the natural geometry of large flakes.

These two reduction sequences share a common unidirectional exploitation and the application of direct percussion. We rarely notice the presence of a lip on the ventral surface nor do we observe grinding of the overhang surface of the butt, which would support the application of a marginal and tangential percussion (see [100]). The butts of blades have a mean width of 13.5 mm and a mean thickness of 4.5 mm while the butts of laminar flakes have a mean width of 17 mm and a mean thickness of 6 mm. These observations suggest the application of an internal (impact point positioned ca. 5 mm behind the edge of the removal surface) percussion with a hammer stone. 12.5% of the blades and 10% of the laminar flakes have a facetted platform.

The assemblage contains a fair proportion of bladelets. The continuous size distribution may indicate that bladelets represent a final stage of the laminar production (Fig 9). However, one bladelet core, in quartzite, shows exploitation with two opposed platforms suggesting these products may partly originate from an independent reduction strategy. Further work will have to clarify which roles bladelets played within the technical and functional system of BRS inhabitants. Indeed, we suppose that the origin of some of these bladelets is related to the sharpening of thick end-scrapers, thus amplifying their proportion within the assemblage. Correspondingly, 97% of the bladelets have a plain platform.

Flakes form the dominant category in terms of technological classification and 8.5% of them present a facetted platform. They vary in terms of shape even though triangular morphologies are rare (2% of the flakes). Flakes originate from the different stages of preparation and management of the laminar cores, as well as from independent reduction strategies. We presently identify two main reduction strategies. The first one is Levallois, which is notably documented by one quartz core that shows centripetal removals on its last stage of exploitation. The second reduction strategy involves the use of an anvil and is documented by one quartz core with adjacent and successive surfaces of exploitation that create a polyhedral form of the core. A few flakes are associated with the use of anvil percussion, but they only represent a minor component of the assemblage (2.5% of the flakes bear typical percussive marks, see [101] for a list of criteria). This bipolar technique of percussion and production exclusively relates to the exploitation of quartz with the only exception of a single quartzite flake.

**Fig 11:**
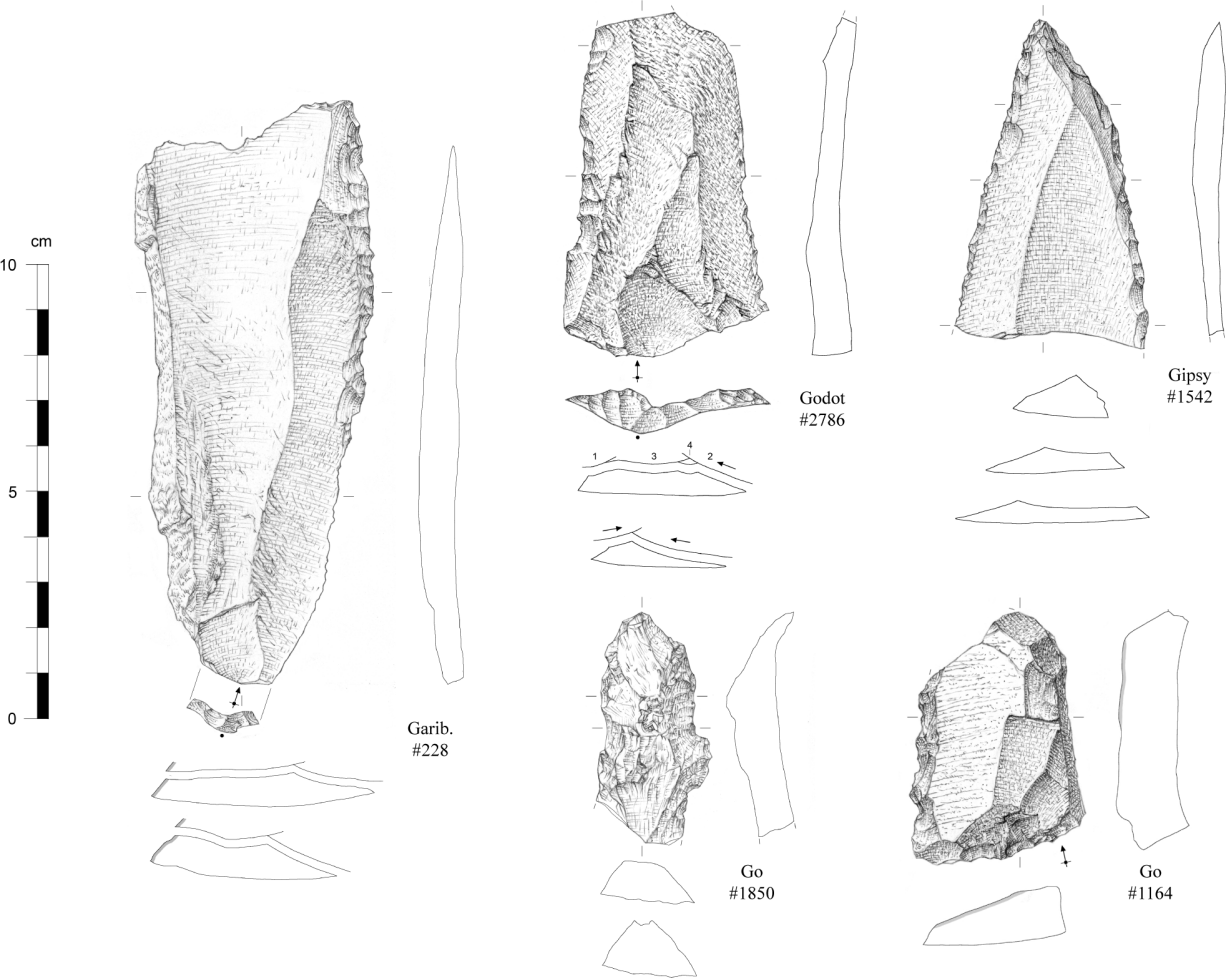
Retouched blanks from the Pietersburg phase ‘28’ of Bushman Rock Shelter (#228 and #2786: Side-scrapers; #1542: Convergent scrapers; #1850 and #1164: denticulates) (drawings by M. Grenet).

**Fig 12:**
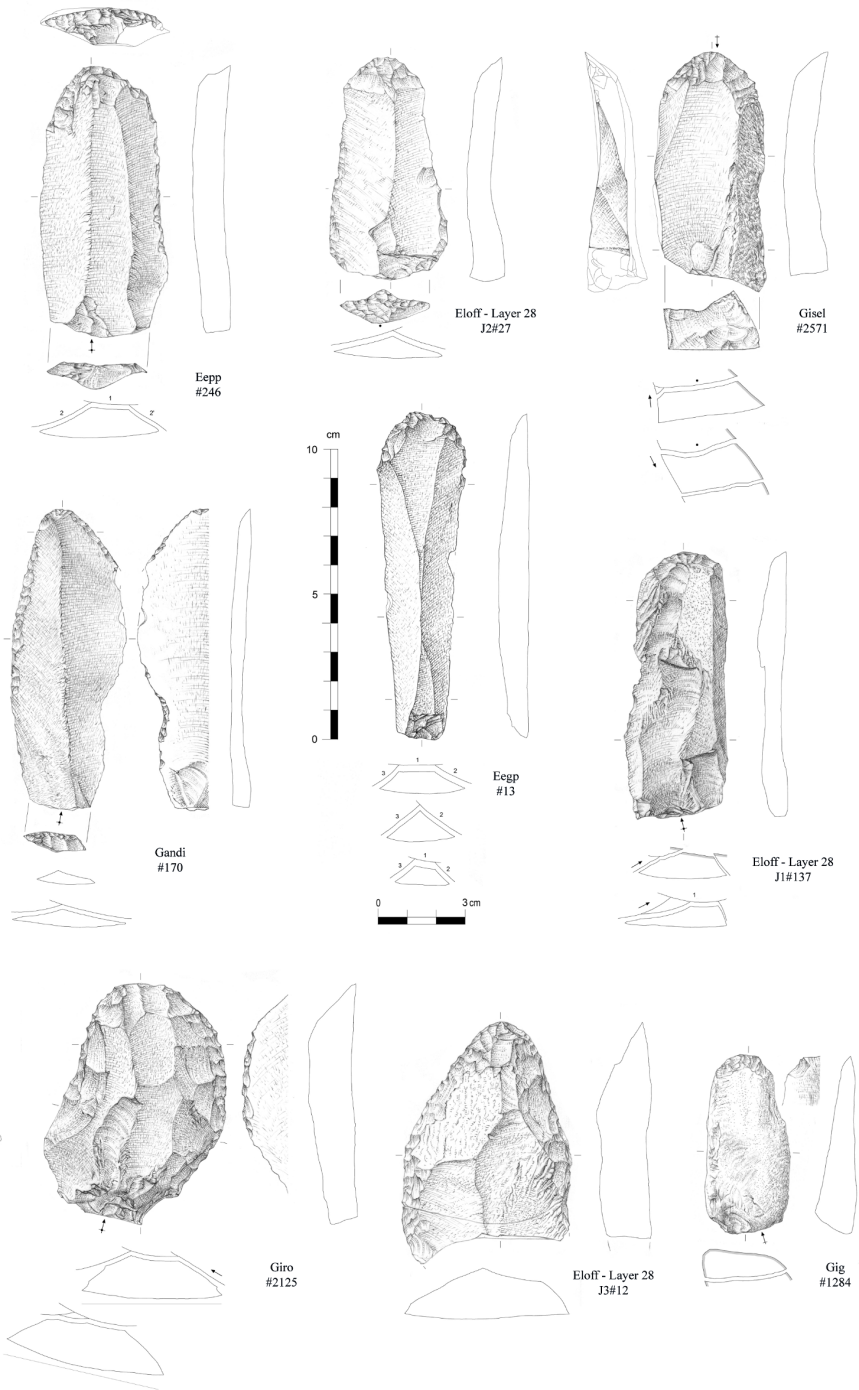
End-scrapers from the Pietersburg phase ‘28’ of Bushman Rock Shelter (drawings by M. Grenet).

**Fig 13:**
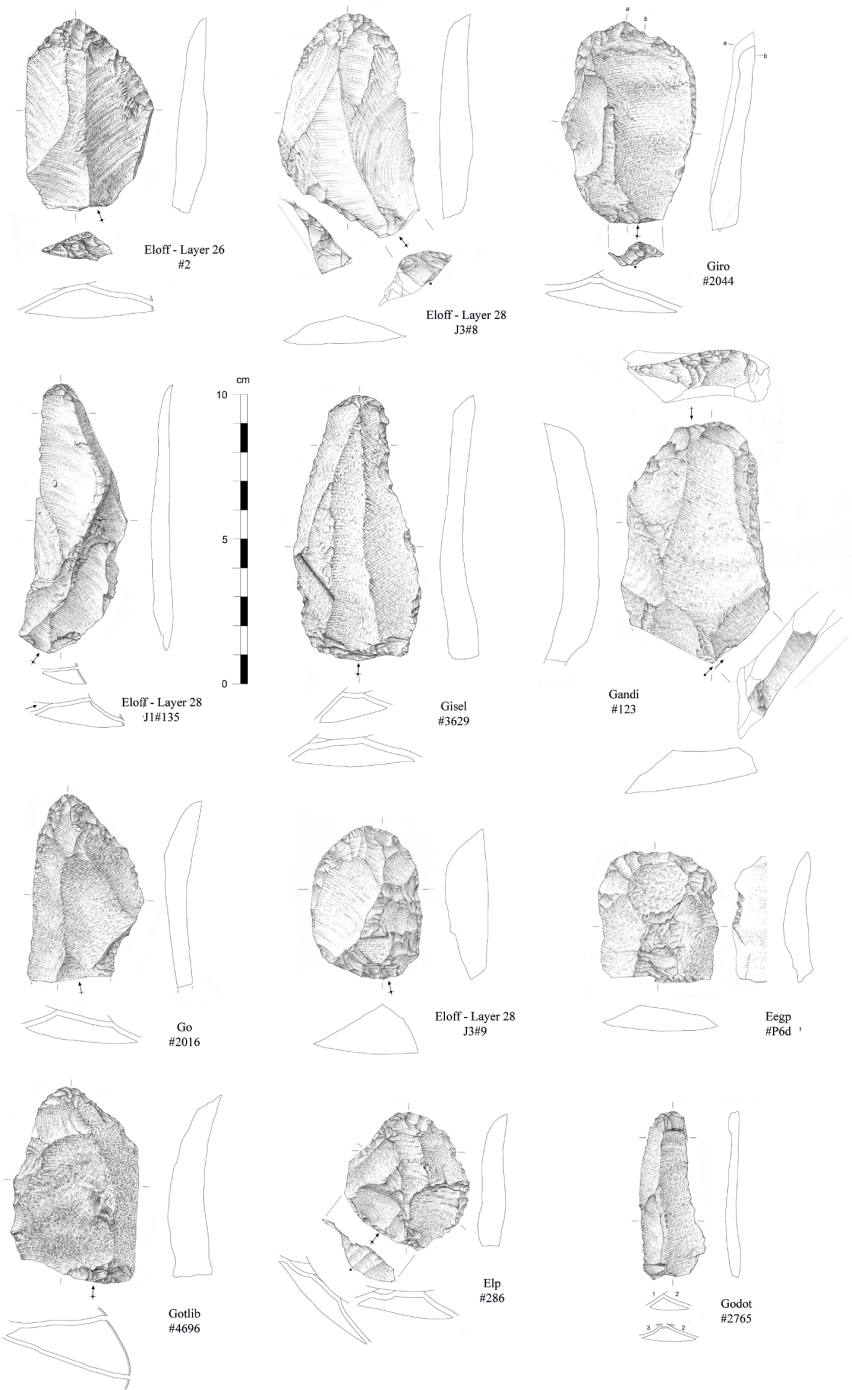
End-scrapers from the Pietersburg phase ‘28’ of Bushman Rock Shelter (drawings by M. Grenet).

Formal tools compose 5.2% of the assemblage and are represented by end-scrapers, side-scrapers and denticulates/notches (Figs 11–13). Convergent forms are rare. End-scrapers have been largely shaped on hornfels (37 on a total of 38 for which raw material could be identified) while notches and denticulates were preferentially made on quartz (11 on a total of 15). While we observe a wide range of products that were chosen for retouch (blades and flakes, cortical or not, *débordant* or not), no bladelets have been transformed. Considering these observations, it is worth noting the discovery of three long bone shaft fragments of non-identifiable large ungulates bearing macroscopic marks possibly documenting their use as retouchers. The lithic assemblage also contains three pebbles, including one in quartzite, with a few percussive marks.

## A focus and discussion on the end-scrapers of the Pietersburg

### Sample

End-scrapers are formal tools that represent blanks with a distal active end (front), which has been retouched into a (sub)convex shape. In the present study, we analysed a total of 131 end-scrapers that were recovered during our 2015 and 2016 excavations (n = 53) and from Eloff’s excavation of layer 28 (n = 78). Our sample includes two specimens with two fronts (each specimen being counted twice); one piece covered by hard sediments was later excluded from our database, reducing our sample number to 130. For micro-wear analyses, the study focused on a sample of 86 end-scrapers, including 40 from our 2015 and 2016 excavations and 46 from Eloff’s collection of layer 28.

In total, our sample includes 87 complete and 43 broken specimens. Due to the selection criteria, proximal parts of broken end-scrapers are not represented. Only two fragments have been refitted, showing an oblique step fracture that may have occurred during use. The spatial distribution of these two fragments indicates limited vertical and horizontal displacement (i.e., same square, same SU, two successive *décapages*).

The pieces present a good state of preservation. We acknowledge, however, some differences between the specimens from our excavation and those from Eloff’s. These differences reflect distinct curation procedures and relate to various degrees of intensity in the way pieces were washed and manipulated. Hence, Eloff’s end-scrapers display recent micro-removals while some of them have been glued and/or partly painted during labeling, all modern marks covering potential technical and functional stigmata.

A few pieces present intense marks of burning or weathering suggesting that end-scrapers have been exposed to various post-depositional processes before and during deposition. Of interest is the fairly high number of pieces with a double-patina (n = 47, i.e., 36% of the studied sample), which for the present study illustrates a difference in surface colour between the retouch of the front and the rest of the piece. This double patina is visible on both hornfels (38% of hornfels) and quartzite (25% of quartzite). On hornfels, the colour contrast is often characterized by a reddish coating that developed on the dorsal and/or ventral piece surfaces. We do not exclude the possibility that this reddish coating represents red pigments that deposited, for example, during ochre processing. However, the fact that some blanks have only a reddish patina on their dorsal surface implies that the alteration of the surface may have occurred on the core before the production of the blank. Additionally, not all patinas are reddish in colour, which may be a further indication of an exposition phase to natural agents (superficial weathering) rather than the result of anthropogenic activities. Though the processes behind patina formation still needs to be clarified, pieces with a double-patina indicate that the manufacture (and use) of end-scrapers was segmented in time, implying period of abandonment and re-use.

### Analytical method

Our study of the end-scrapers combines three analytical steps. The first step is technological and aims at characterizing the different stages of manufacture of the end-scrapers and at discussing how this fits within the global technological system. In sum, what were the criteria of selection with regard to the raw materials, morphologies and dimensions of the blanks?

**Fig 14:**
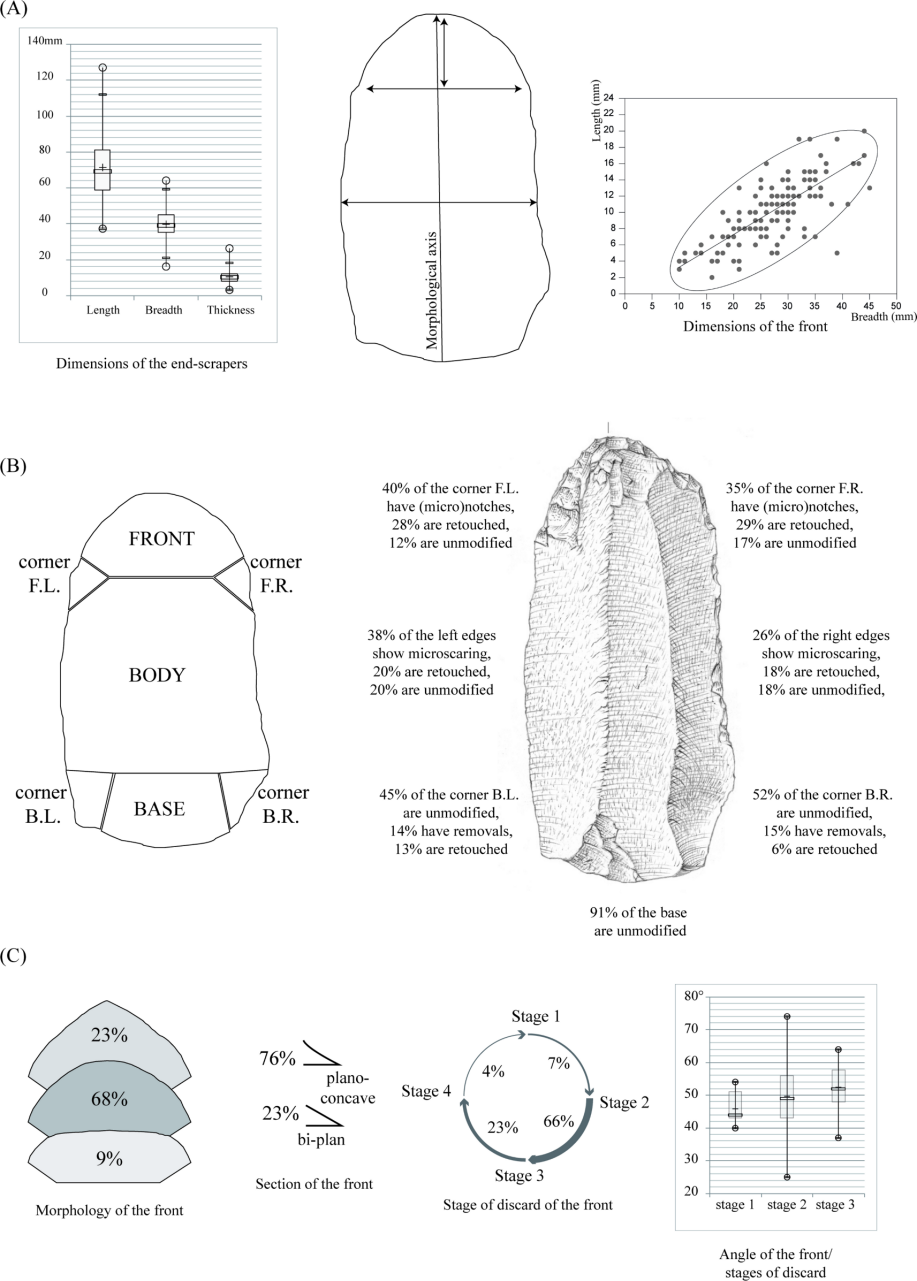
**Synthetic figure of the end-scrapers from the Pietersburg phase ‘28’ of Bushman Rock Shelter:** (A) dimensional variability, (B) distribution of the technical and macro-functional scars and (C) stages of discard.

The second analytical step is techno-functional, aiming at describing how the tool is structured [102] and what is the significance of their morphological and dimensional variability. All end-scrapers have been orientated within their morphological axis (perpendicular to the front) and described following a subdivision into three main parts that are (1) the front, (2) the body and (3) the base. In addition, and assuming that these parts may bear specific traces, we described separately the corners of the *front* (subsequently called ‘corners-F’) and those of the base (subsequently called ‘corners-B’) (see Fig 14B). All these parts have been characterized with regard to the intentional (technological) and unintentional (functional) traces they were bearing. Attention has been paid to the location, distribution, morphology and dimensions of all the different macro- and micro-removals at a low magnification (x10-x40). Fronts have been additionally described by considering their section, angle, morphology and dimensions. We also ascribe each front to a ‘stage of discard’ that we defined as follows: stage (1) (‘in use’) when the front edge is fresh and without irregularities, stage (2) (‘to be sharpened’) when the front edge has several macro- or micro removals related to function, stage (3) (‘in resharpening’) when the front has intentional but discontinuous removals, and stage (4) (‘out of use’) when the front is broken.

The last analytical step is functional and intends to identify how and on which raw materials end-scrapers were used. The functional study rests on the combination of macroscopic edge damage features together with microscopic use-wear traces (see [103,104]); it also includes the recording of macro-residues when visible on the surface of the tools. These modifications are visible under low magnifications as scars, fractures, edge rounding, and under the microscope as polishes, striation and micro edge rounding. All artefacts were examined following the standard procedures applied in use-wear analysis [105,106] and with equipment as described in Igreja [107] and Igreja and Porraz [108]. Some of the end-scrapers have also been studied for their micro-residues (analysis in progress at the University of Liège, Belgium).

### Technological and morpho-metric description

The end-scrapers are made on hornfels, which compose 92% of the total collection, with a clear preference towards fine (37% of hornfels) and medium qualities (61% of hornfels). The only other rock represented is fine-grained quartzite. Considering that our sample is largely dominated by one rock type, we present our results regardless of the nature of the lithic raw materials.

The tools have a mean length of 74 mm, a mean width of 40 mm and a mean thickness of 10 mm. There is an important dimensional variability (Fig 14A), firstly reflecting in the length of the end-scrapers that extends from 37 mm to 127 mm. However, the length distribution may support the existence of one single population (Fig 14A). Several factors have to be considered when elaborating on the variability of the end-scrapers length. First of all, the intensity of tool reduction directly impacts the longitudinal axis of the tool. Secondly, the nature of the blanks selected to manufacture the end-scrapers has to be considered as well.

Though the LI is 1.8, the technological description of the blanks indicates that most of the blanks were blades (55% of the sample) and laminar flakes (30% of the sample). This implies that (1) the manufacture and shaping of the end-scrapers substantially impacted the length of the original blanks and (2) the mean dimensions calculated for the end-scrapers should be regarded as a minimal size before discard. End-scrapers have been preferentially manufactured on elongated blanks, but we acknowledge a selection occurring at different technological stages and orientated towards various morphological attributes. We observe a selection that includes *entames* (primary blanks) and cortical blanks (12.5% display a surface with >10% remaining cortex), *débordant* blanks as well as Levallois products. Of interest is the fairly high percentage of facetted platforms that amounts up to ca. 45% of our sample (n = 75 end-scrapers with a preserved butt). In terms of morphology, the end-scrapers have been manufactured dominantly on symmetric blanks with parallel (64% of the blanks) or convergent edges (26%). Though blank sections may vary (symmetric or asymmetric), they all present a straight profile (83%).

### Techno-functional characterization

End-scrapers’ fronts have been manufactured on the distal part of the blanks (92% of the sample), predominantly within their percussion axis (with a 10° variation). However, we notice a high proportion (42%) of end-scrapers with a morphological axis that is off-centered from the technological axis. This off-centered character shows no preferential orientation (either right or left to the axis of percussion) and varies within an angle bracketed between 11° to 30°, with rare exceptions going up to 65°.

Most of the end-scrapers’ bases (91% of the sample) have been left unmodified, while their corners-B present a modification in ca. 20% of studied cases (Fig 14B). These modifications take the form of a retouch or of one to two removals that have been detached from the ventral face, following the delimitation ridge of the platform. While the retouch corrected the general delineation of the corners, the removals on the ventral face thinned the proximal part of the tools.

The edges of the end-scrapers have been left unmodified in more than 80%. Twelve end-scrapers present a retouch on both of their edges, while 10 end-scrapers present a retouch on only one edge. The retouch of the edges presents a substantial variability with regard to their angles, sections, lengths, delineations and invasiveness. While some end-scrapers show a marginal modification of their edge(s), others document edges with modifications that suggest they have been reworked several times.

The width of the fronts shows some variability that is directly related to the morphologies and dimensions of the blanks that have been selected. The front width is bracketed between 10 to 45 mm, with a mean of 27 mm (Fig 14A). While the dimensional distribution is continuous, suggesting the existence of a single population, we are, however, inclined to propose the existence of several functional populations, considering that the width of the front forms the extension of the contact zone whilst the tool is in use. We observe that convergent blanks are twice as many in the population of end-scrapers with a width below 22 mm compared to the whole population, suggesting that the selection of these morphologies could be associated with a specific functional intent orientated towards narrow fronts.

In terms of morphology, the fronts dominantly show a regular convex shape (68%), while 9% tend to be rectilinear and 23% pointed (Fig 14C). The variability of the fronts, also illustrated by their length (Fig 14A), seems to partly relate to the stages of discard. The fronts document a stage 1 (“in use”) in 7%, a stage 2 (with functional scars) in 66%, a stage 3 (with technical stigmas) in 23% and a stage 4 (broken front) in 4% of the studied cases. Only a few end-scrapers (n = 9, classified within the stage 1) present a front that has not been substantially modified by use and, interestingly, they all represent a convex morphology, with one exception being a pointed morphology. In that regard, we suggest rectilinear morphologies may represent a stage of intense reworking rather than an intentional shape. Interestingly, the mean length of rectilinear fronts (4.5 mm) is substantially smaller than the rest of the sample (10.8 mm).

The importance of sharpening is further illustrated by the functional characteristics of the fronts. While their sections show stable characters (76% of the fronts have a general plano-concave section and 23% have a bi-plan section), their angles vary more substantially though 75% of the sample document angles between 45° and 55°. Nevertheless, of interest is the relationship between the stages of discard and the angles of the front, as we notice a gradual increase from stage 1 (mean angle of 46°) to stage 2 (50°) and stage 3 (52°) (Fig 14C). In addition, measurements associated with stage 2 document a wider range of angles than for stage 1 and 3.

Finally, attention should be paid to the corners-F that have been modified in ca. 40% of the studied cases (ca. 20% of the end-scrapers have a modification on only one corner-F, and ca. 20% show modifications on both corners-F). These modifications dominantly take the form of an intentional (micro-) notch adjacent to the front, which has been observed in 70% of the corners-F that have been modified. Alternatively, the corners-F have been modified by a retouch localized in the continuity of the front. Additionally, we identify some specimens with a front that has been shouldered (on one side: n = 3, on two sides: n = 3), which may be considered as an adaptation intending to facilitate the shaping and/or to control the dimensions of the front.

### Results of the use-wear analysis

The set of use-wear traces allows us to elaborate on three different functional categories that are: the used areas, the raw materials that have been worked, the way the tools were used and handled. While we recorded macro-traces on our whole sample of end-scrapers, the micro-wear analysis focused on a corpus of 86 tools. Within this sample, micro-traces have been identified on 33 end-scrapers, recording a total of 43 used zones (Table 10): 5 artifacts show evidence of use on both the front and an adjacent edge. If we exclude the category of “weathered pieces”, the percentage of analysed tools that show micro-wear traces amounts up to 47%.

**Table 10:** Use-wear synthesis for the studied end-scrapers from the Pietersburg phase ‘28’.

						WORKED MATERIALS AND MOTIONS										
		STATE OF PRESERVATION		With	Number of Used	Wood	Hard materials			Mineral		Bone		Animal Soft materials		
	Total	good	weathered	USE-WEAR	ZONES	scraping	cutting	scraping	hafting	cutting	scraping	scraping	cutting	cutting	scraping	Total
End scrapers 2015–16	40	25	15	14	21	4	3	7	-	-	-	4	1	-	2	21
End scrapers "Eloff"	46	45	1	19	22	-	2	8	1	1	9	-	-	1	-	22
**Total**	**86**	**70**	**16**	**33**	**43**	**4**	**5**	**15**	**1**	**1**	**9**	**4**	**1**	**1**	**2**	**43**

First of all, we notice that use-wear traces largely concentrate on the front (34 used zones on a total of 43). These traces take the form of small spots of polished surfaces, sometimes in association with striations. The nature and development of the polishes indicate that the front of the end-scrapers exclusively worked in a longitudinal motion, the front being used in a transversal contact with the raw material such as in a scraping activity.

**Fig 15:**
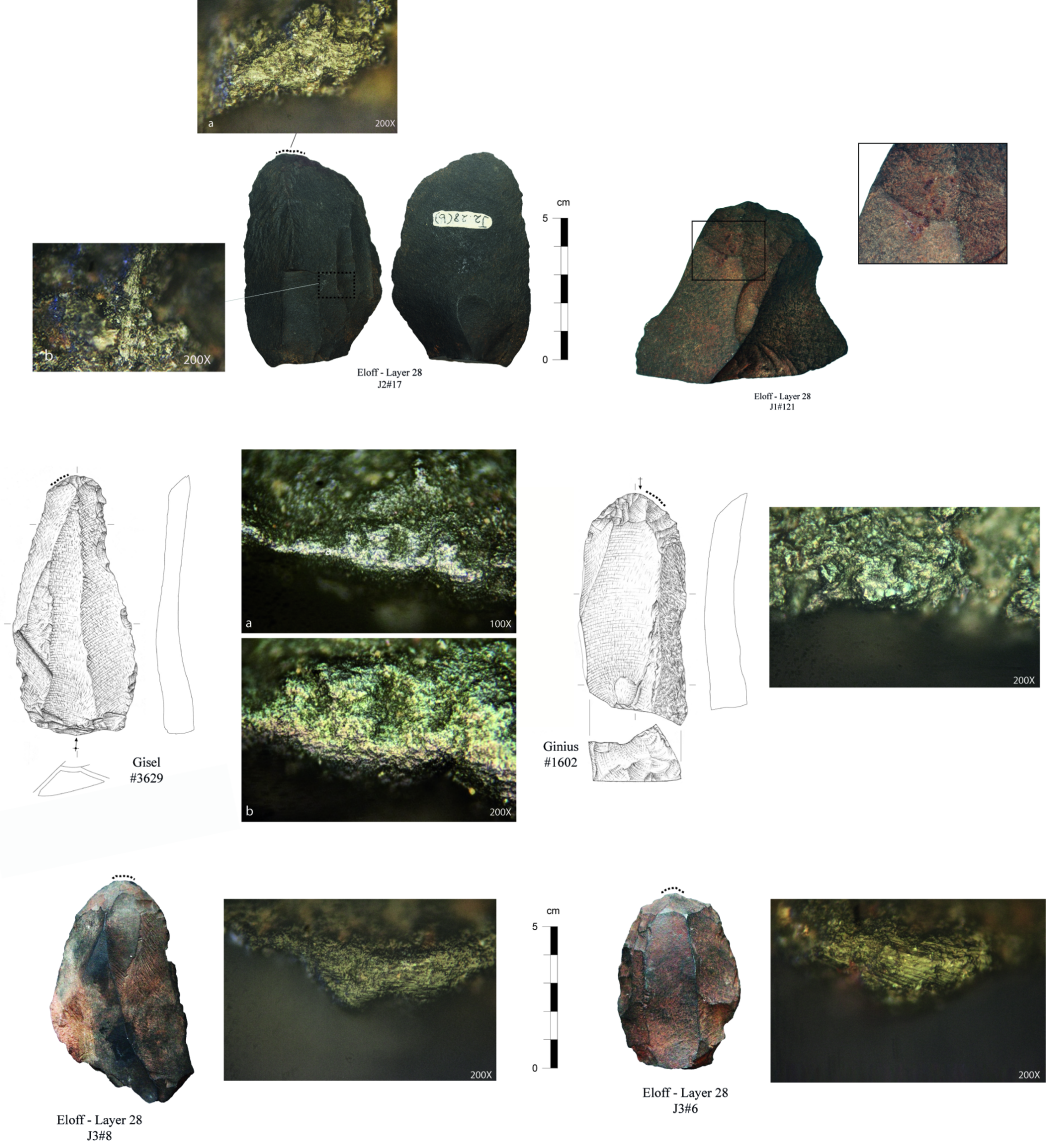
Use-wear and residues on the end-scrapers from the Pietersburg phase ‘28’ of BRS (#17: on the front, polish characteristic of mineral material scraping; on the dorsal ridge, polish possibly related with hafting. #121: blackish organic residue possibly the remnant of an adhesive. #3629 and #1602: polishes characteristics of hard material scraping; #8 and #6: polishes characteristics of mineral material scraping) (pictures by M. Igreja).

**Fig 16:**
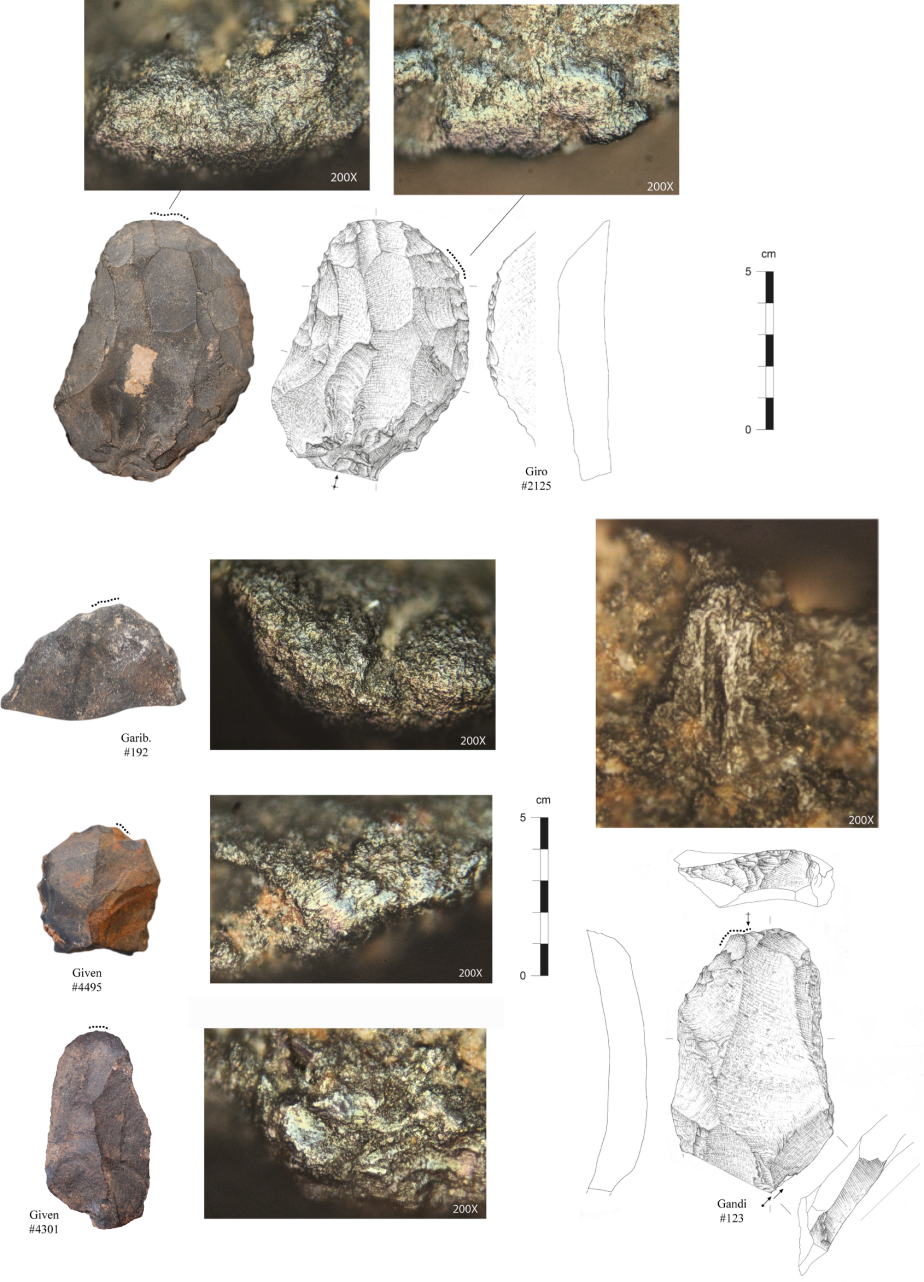
Use-wear on the end-scrapers from the Pietersburg phase ‘28’ of BRS (#2125 and #182: polishes characteristics of dry hide scraping; #4494 and #123: polishes characteristics of bone scraping; #4301: polish characteristic of wood scraping) (pictures by M. Igreja).

These raw materials were of various natures, but predominantly with hard properties, as documented by polishes that are bright and smooth in texture, gently curved over the high points of the microtopography and distributed along the edge in a reticular pattern (Fig 15). Only two end-scrapers show a front with a highly developed edge rounding and matt texture, polish that is suggestive of a contact with a soft material such as dry hide (Fig 16). When determinable, the hard raw materials are represented by wood (n = 4), bone (n = 4) and minerals (n = 9). It is presently unclear why mineral polishes are exclusively associated with Eloff’s collection and how they formed. The extension of the traces does not support the hypothesis that the polish formed by abrading the edge before shaping (operation that would have intended to isolate and reinforce a contact point before percussion). While the polish could relate to an intense contact with a rich mineral load, the intensity of the traces suggests that the front of the end-scrapers may have worked directly in contact with a soft mineral.

At a macroscopic scale, the fronts in general often display isolated unintentional micro- and macro removals located on the ventral face. A few of these removals are very large (with a maximum length of 9 mm and a maximum width of 16 mm) implying that it was caused by an axial and percussive force. It is unclear whether these large removals reflect a last stage before discard, engaging the tool in a different motion than the one it was intended for. However, their frequency corroborates the hypothesis that end-scrapers were involved in activities implying work with/on hard materials.

Various lines of evidence help inferring the way tools were manipulated. The transversal motion of the tool, as deduced from the striations on the front, provides indications on the kinetic of the end-scrapers, but the way they were handled remains unknown. We have very little evidence of hafting. One clue is provided by a large end-scraper (Fig 15) that shows intense macro-polishes on the proximal part of the tool, both on the dorsal and ventral faces, suggesting a repeated contact and friction with a hard material. Additional indirect evidence can be drawn from the presence of a potential macro-residue located nearby the front of a broken end-scraper (Fig 15). This residue has a blackish organic colour and an irregular texture that has not been observed on the surface of other pieces or in the sediments. This residue resembles what has been described on artefacts from other MSA and LSA contexts in southern Africa (e.g., [109–111]) and may represent the remnant of an adhesive, but further analysis is required.

The way the end-scrapers fractured can be additionally used as a proxy to infer the extension of the haft and/or its morphology. On a total of 43 broken pieces, we note a clear dominance of straight fractures perpendicular to the axis of the tool (45% of the fractures) while oblique (28%) and bending fractures (14%) also characterize our sample. The range of fractures is associated with various origins (during sharpening and in different motions), but also indicates that the fracture, which occur during use, involved some force and, consequently, a solid system of handling. Interestingly, we notice very few “broken fronts” (n = 13), i.e., fractures that initiate in or very close to the corners-F, fragments that are often interpreted [112] as indicative of the limit of the handle (regardless of the origin of the fractures). Though the lack of the proximal part of broken end-scrapers is prejudicial to the general interpretation, the rarity of broken fronts may indicate that the limit of the “handling system” was not located close to the front. The frequent removals observed on the corners-F (Fig 14B) are further indirect evidence of the hafting of end-scrapers, although their origin (bending?) remains to be clarified.

This functional analysis provides evidence that the edges of the end-scrapers were also occasionally used. In total, five tools present micro-wear traces suggesting a use in a longitudinal motion. These edges were used retouched or unmodified, and mostly involved in the working of hard materials such as bone and mineral. It is presently unclear to state on the nature of the functional relationship between the front and the edges. Either the edges were active contemporaneously to the front (implying some adaptations in terms of handling/hafting strategies) or they were active time-independently.

### The end-scrapers of BRS: A synthesis

Our sample provides a first overview of the main attributes of the Pietersburg end-scrapers and of their degree of variability. Furthermore, the functional study allows us to elaborate on the way these tools were manipulated and for which activities they were intended to be used.

Our first remarks regard raw material economies [113]. We observe a clear preference towards hornfels for the manufacture of end-scrapers and, concomitantly, a non-selection of quartz while this rock composes 20% of the whole lithic assemblage. This discrepancy does not relate to dimensional attributes as quartz and hornfels artefacts do not vary substantially with regard to their mean length and width (Fig 9). One line of explanation could relate to the physical properties of the respective raw materials, considering that quartz is a harder rock than hornfels (respectively 7 and 2–3 on the scale of Mohs). Such difference, according to the intended tasks, likely influenced the patterns of rock selection and transformation.

The analysis of the blanks highlights the selection of a wide range of blank types, in terms of technical characteristics as well as in terms of dimensions and morphologies. However, our study points towards a general pattern of selection that favoured elongated blanks (blades and laminar flakes), with straight profiles and rectangular or to some lesser extent triangular morphologies. Considering all blanks of the lithic assemblage, the blanks selected to be transformed into end-scrapers present a higher proportion of platform preparation (45% of the end-scrapers have a facetted platform while only 9% of the laminar blanks are facetted), suggesting that the knappers paid special attention during their production.

We observe a certain degree of flexibility in the way the end-scrapers were manufactured, starting with the general orientation of the tool axis that diverges from the technological axis in more than 40% of the studied cases. We also identify some variability in end-scrapers dimensions as well as in the dimensions and characteristics of the fronts. However, this variability disguises a simple and normalized structure of a tool that was organized around its distal active edge. The blanks were occasionally modified but most of the manufacture was devoted to shape a *front* with a convex to (sub)pointed morphology, a plano-concave section, an angle between 40° to 50° and two corners that were notched or retouched.

Apart from technological criteria, we recognize two main factors explaining the variability of our sample. The first one relates to the intensity of use and sharpening, which is illustrated by: 1) the LI of the end-scrapers (1.8) while they have mostly been manufactured on blades and laminar flakes, 2) the stages of discard of the front, which suggest that the tools have been intensively used, and 3) the fairly high proportion of end-scrapers with use-wear (42% of end-scrapers). The second factor relates to recycling; this is supported by: 1) numerous specimens with a double patina indicating that the tools and/or the blanks were discarded and re-used several times and 2) several specimens that have also been used with their edges (retouched or not) in a longitudinal motion. The chronology of tool recycling is unclear; in other words, whether used blanks were selected to manufacture end-scrapers and/or if end-scrapers were recycled to be used as knifes. We interpret the data within a model that echoes a general pattern including blanks that were used -previously, simultaneously or afterwards- in other actions and with different active parts. Such observations indicate some time lags between the production of the blanks and the (re)use of the tools. It suggests a “long life” for the tools that were used, discarded and recycled.

The functional study shows that end-scrapers were intended to work different raw materials, hard (such as bone, wood and mineral) or soft (such as hide). The results of the study indicate that end-scrapers were not a specialized tool in terms of function, but in terms of motion. End-scrapers were strictly working in a transversal motion with a front that was used to scrape the raw material, either to thin it, soften it or to produce powder.

## The Pietersburg techno-complex at BRS in the context of the southern African Middle Stone Age

### Context and new results from BRS

The present paper discusses the upper MSA occupations at BRS based on renewed excavations carried out in 2015 and 2016. Due to the geometry of the remnant deposits, our excavations of the MSA deposits started at the base of layer 27. They aimed at exploring what we informally and temporally named the phase ‘28’. With regard to past excavations, we clarified the following points:

1. The stratigraphic succession: unlike previous field observations, we noted that the layers composing the phase ‘28’ are not laterally continuous but form two main depositional phases that are associated with, respectively, our SUs ‘Go-’ and ‘Gi-’. A total of 12 SUs has been subsequently recognized and correlated with the layers 28 to 30 of Eloff.
2. The site formation processes: the geoarchaeological study documents a significant anthropogenic input in the depositional processes, dominantly taking the form of ashes, with local concentrations of roof spalls that can be explained by the proximity to the shelter wall. The results suggest that hearth-related activities were an important component of the daily life activities at the site. It indicates that the back of the shelter was not only a dumping area but was part of a broader living space.
3. The chronology: to refine the chronology of the occupations, four OSL samples have been collected on the south profiles and analyzed with respect to their quartz and feldspar content. Two samples (BRS1 and BRS2) come from the LSA deposits for which we already benefited from a good set of radiocarbon dating, and two samples come from the upper MSA deposits, namely from the Pietersburg phases ‘21’ (BRS3) and ‘28’ (BRS4). The luminescence technique provides two sets of ages for BRS3 and BRS4, dated respectively to 73±6ka and 75±6ka on quartz and to 91±10ka and 97±10ka on feldspar. Despite the uncertainty, we can make the following statements: (1) the hiatus between the LSA and the MSA occupations characterizes a minimum interval of time of 46,000 years, (2) no MSA occupations postdate the MIS5 at BRS, and (3) the Pietersburg of BRS (phases ‘21’ and ‘28’) represents a period of time (at one sigma) that lasted for a maximum of 14 000 years based on quartz OSL or of 26 000 years based on feldspar OSL.
4. The archaeological collections: our excavations unearthed a diverse set of mineral and organic remains, including lithic artefacts, ochre pieces, fauna remains, charcoals and seeds. These remains vary substantially in terms of density and frequency, reflecting certain variability in the accumulative processes and the nature of the activities performed at the site. Though a few beads were discovered by Eloff in the phase ‘28’ [54], our excavation did not yield any new beads nor OES fragment. Direct dating of the beads will have to clarify their origin.

### The Pietersburg lithic technology

In 2015 [51], we published a preliminary overview of the lithic assemblages from layers 21 to 24 (informally and temporally named phase ‘21’). The lithic assemblages from those layers are composed of two dominant rock types, namely hornfels and quartz, which were reduced following similar reduction sequences. The objectives of the knappers were orientated towards the production of various blanks including (large) blades, laminar flakes and flakes. Although the lithic assemblage was not studied in detail, we recognized the coexistence of several reduction strategies including a Levallois method and a (semi-)prismatic blade method. One striking character of these lithic assemblages is reflected in their typological corpus, which includes tools that were shaped unifacially on their ventral face or bifacially (including bifacial pieces and bifacially retouched pieces).

This paper focuses on the lithic assemblages from the phase ‘28’. We notice that hornfels forms the main raw material while quartz is the second raw material selected by the BRS inhabitants. The reduction sequences were largely orientated towards the production of laminar blanks, following a unidirectional Levallois method and a semi-prismatic reduction strategy. Typologically, the formal tools are typified by the presence of end-scrapers, but also include side-scrapers, notches and denticulates.

The contact between the phases ‘21’ and ‘28’ at BRS requires to be verified by renewed excavations. In particular, it is yet unclear whether the coexistence of end-scrapers and bifacial pieces, as documented in the layers 24 and 25 of Eloff, represents a real typological trend or is only due to excavation biases. However, despite this typological difference, these two phases share similarities in terms of their pattern of raw material selection and the nature of their reduction strategies, suggesting to us that they share a similar technological background.

We assigned the phases ‘21’ and ‘28’ of BRS to the Pietersburg, following previous publications and descriptions [29,38,39]. Nevertheless, we notice some inconsistencies in the way this techno-complex has been previously characterized as well as some differences between the BRS lithic assemblages and those previously assigned to the Pietersburg.

Using the lithic artefacts from Cave of Hearths as a basis, Mason [38] subdivided the Pietersburg in three main stages: Bed 4 represents Early Pietersburg, Bed 5 Middle Pietersburg and Beds 6–9 Late Pietersburg. These phases include laminar products (classified as ‘*long quadrilaterals*’) and triangular flakes (classified as ‘*short triangular*’). The definition of the three stages is based on substantial variations from earlier to later stages with a decreasing proportion of laminar products (from 41.8% to 24.1% of ‘*primary classes*’) and an increasing proportion of triangular flakes (from 3.4% to 15.8%). The presence of a Levallois reduction strategy is not formally stated by Mason but the data and the published illustrations [29] allow us to hypothesize the application of a unidirectional Levallois method (see also [114]). Of interest may be the fairly high percentage of faceted-platforms (from 37.5% to 60.1% of the ‘*primary class striking-platform*’) and their increasing proportion from earlier to later stages [29]. Finally, formal tools help in differentiating these three stages, as a higher proportion of end-scrapers is noticed in the middle stage and a use of bifacial shaping is strictly limited to the later stages.

Cave of Hearths certainly is the key-site for defining the Pietersburg, and together with Mwulu’s Cave, Olieboomspoort, Koedoesrand and Aasvoëlkop, formed the basis that Mason [29] used for comparison. Of particular interest are the lithic collections of Mwulu’s Cave published by Tobias [39]. The excavation allowed him to recognize three distinct layers with lithic assemblages that show ‘*characteristic prepared platform and convergent longitudinal flaking*’ ([39]: 5). On the basis of the description and illustrations, we are tempted to recognize the use of the Levallois method while faceting of the platforms was frequent (respectively 46%, 49% and 42% of the ‘*primary class striking platform’* in [38]). Of interest is also the typological description provided by Tobias who noticed ([39]: 10) ‘*The more advanced phases show a high degree of secondary trimming on the upper surface of flakes*, *butt and bulb-reduction and further trimming on the flake-surfaces*, *ranging in degree up to the most elaborate bifaced forms*’. Together with the unifacial and bifacial points occur several “*end-scrapers and end- and side-scrapers*” [39].

Present morphological and typological descriptions tend to clearly differentiate the later stage of the Pietersburg from its early expression. This is firmly stated by Mason who advances the existence of ‘*outstanding differences*’ ([38]: 132) between the earliest and the last stage of the Pietersburg and who acknowledges that ‘*the general change in terms of the ten factors of analysis is greater from the earlier stage to the middle stage than from the middle to the later*’ ([38]: 135). However, the study of Sampson [29] introduces some confusion with contradictory results, showing that the lithics from Bed 4 and Bed 5 of Cave of Hearths are directly related, while those from Beds 5 and 6–8 change abruptly: “*it appears reasonable to assume that Beds 4 and 5 represent an earlier and later phase of the same cultural (or technical) tradition*” ([29]: 93). Further confusion arises from the assignment of the upper MSA at Border Cave to an ‘Epi-Pietersburg’ industry ([45], but see [43]), which is said to contain a few bifacial pieces.

The attributes allowing us to assign the phases ‘28’ and ‘21’ of BRS to the Pietersburg are the presence of laminar elements -including a Levallois production-, and of peculiarities within a typological corpus comprised of end-scrapers and unifacially or bifacially shaped points. It is currently difficult to discuss the validity of the three different stages as recognized by Mason [38]. Based on our description, the sequence of BRS provides assemblages that are reminiscent of the Late and Middle Pietersburg, while the preceding phase ‘36’ differs substantially in terms of technology and typology.

We distinguish two Pietersburg phases at BRS that we individualize as lower (‘28’) and upper (‘21’). The recognition of these two phases is notably supported by the absence of interstratifications between the main tool categories (no bifacially retouched pieces occur below the layer 25, no end-scrapers above the layer 24), though these tools may coexist in some lithic assemblages (as noticed at Mwulu’s, [39]). One main difference that requires clarification regards the triangular flakes, which are seldom in the ‘28’ and ‘21’ phases of BRS while they contribute to the definition of the Pietersburg by Mason [38]. How much did subsistence strategies and site activities impact the distribution of Pietersburg lithic attributes will have to be explored in future as well.

### The Pietersburg within the set of MIS5 lithic technologies in southern Africa

Luminescence dating at BRS places the Pietersburg within the MIS5, with occupations bracketed between ca. 100ka and 75ka. While over the last decade, the interest for MIS5 lithic assemblages has increased, a few sites only have recently been (re-)investigated. Such sites are, with a couple of exceptions, mostly located in the Western and Eastern Cape Provinces of South Africa.

A recent publication by Douze et al. [24] summarizes the main constitutive elements of MIS5 (pre-Still Bay) lithic technologies of the South African Western and Eastern Cape using the sites of Klasies River Mouth (MSA II, [23,31]), Blombos Cave (M3 phase), Pinnacle Point Cave 13B [115] and Diepkloof Rock Shelter (MSA-Mike, [11]) as references. The authors emphasize a combination of different core reduction methods, with a dominant parallel (Levallois-like) core reduction method, to produce predetermined blanks, notably blades (in variable proportions) and triangular flakes. Formal tools always compose a low percentage of the assemblages and are dominated by notches and denticulates. We may add to this description some data on the MSA-Lynn from Diepkloof Rock Shelter, which represents one single SU that is considered transitional between the MSA-Mike and the Still Bay [11,116]. The MSA-Lynn characterizes a change in raw material provisioning strategies that accompanies the appearance of convergent tools in the sequence, including a few bifacially retouched specimens.

The site of Apollo 11, located in southern Namibia, contains occupations dating to the MSI5, which have been initially classified as Apollo 11-MSA 2 and MSA 1 [117]. The MSA 2, later assigned with some reserve to the Still Bay [118], has been dated by OSL to 71±3 ka, while no direct dating could be obtained for the lowermost layers. The MSA 2 is characterized by the production of blades that are present in high proportion while the formal tools, in addition to four bifacial pieces, include unifacial points, side-scrapers as well as end-scrapers. These last tools comprise a total of nine specimens that represent “*basal end-scrapers on pointed blades*” ([118]: 197). The illustrated pieces document standardized tools that were shaped on normalized blanks and are striking by their homogeneity. These end-scrapers are documented in other Namibian assemblages [117] and are considered as a specific type form by the author, though the functional nature of the front requires some clarification (front to be used or modification for handling purpose?). The MSA 1, individualized in two phases, is characterized by blades and blade-like flake manufacture, though the products are said to be less regular than in the MSA 2. The typological corpus is poor and includes some scrapers and denticulates in the upper phase and a few scrapers, denticulates, notched pieces and truncated blanks in the lower one.

The site of Sibudu Cave, located in KwaZulu-Natal, South Africa, also contains MIS5 informally called ‘pre-Still Bay’ deposits (‘serrates’ layers), which have been partly reported in Rots et al. [2]. The authors briefly describe a laminar industry together with asymmetric convergent tools, scrapers and bifacial pieces. The presence of bifacial implements may be reminiscent of the late Pietersburg although the assemblage from Sibudu Cave includes serrated pieces that are presently regionally confined. The published illustrations of the cores [2] are not consistent with a Levallois reduction strategy.

In comparison with other MIS5 sites, especially those documented in the Western and Eastern Cape Provinces, the Pietersburg of the upper MSA at BRS differs due to the nature and the coexistence of two laminar reduction sequences, the rarity of triangular flakes and the characteristics of its typological corpus. Regardless of the driving factors behind these differences, we observe a certain degree of homogeneity between the sites from the Western and the Eastern Cape Provinces (variously assigned to the MSA 2) on the one hand and the sites from the Limpopo Province (assigned to the Pietersburg), suggesting that these provinces reflect indeed different technological traditions.

Finally, it is worth remembering the presence of end-scrapers in the Still Bay of Blombos Cave [13], as well as in the MSA 2 (*sensu* [117]) of Apollo 11, although the functional nature of the basal end-scrapers from Apollo 11 requires further investigation. These two sites have been dated to a similar time period, which is also consistent with the BRS chronology based on quartz luminescence (73±6ka and 75±6ka). The adoption for the first time of this new tool category, tentatively during the same time interval, could point towards some level of connectedness between human groups from those regions or, alternatively, could be the indication of some technological convergence. The lithic assemblages of Blombos Cave, Apollo 11 and BRS document technological differences, even though there are similarities between Apollo 11 and BRS with regard to the dominant manufacture of blades. Interestingly, while these sites are characterized by the presence of end-scrapers, the way these tools were manufactured differs. Hence, at Apollo11, end-scrapers were manufactured on the proximal part of regular blades (for the confection of an active or passive part); at Blombos Cave, end-scrapers were manufactured on flakes; and at BRS, end-scrapers were dominantly manufactured on the distal part of blades. These differences would illustrate the adoption of a similar typological innovation in contexts that were technologically distinct.

## Conclusion

The study aimed at providing a technological and chronological context for the upper MSA occupations at BRS, a site located in a province that benefited from little attention in comparison to recent work conducted in South Africa. Unlike the Western and the Eastern Cape Provinces, the Limpopo Province provides a window towards the northern and eastern part of southern Africa, contributing to the amalgamation of a wider archaeological and palaeoenvironmental record and to the elaboration of scenarios at a broader geographic scale.

Based on published data, we affiliate the upper MSA lithic assemblages of BRS (phases ‘28’ and ‘21’) to the Pietersburg techno-complex with luminescence dating that placing it during the MIS5. The Pietersburg technology is based on the production of laminar blanks, including large blade specimens, obtained by unidirectional Levallois and semi-prismatic reduction strategies. As presently documented at BRS, the Pietersburg is composed of two main typological phases that are the lower phase ‘28’ with end- and side-scrapers and the upper phase ‘21’ with unifacially and bifacially shaped pointed forms. The modes of succession of these two phases and their significance are questions that still require clarification.

Comparisons with published lithic assemblages from southern Africa show differences that allow us to hypothesize the existence of distinct techno-typological traditions and adaptations during the MIS5. The putative existence of groups that were technologically separated during the MIS5 is of importance with regard to the florescence of innovative behaviours that characterize this period and this region of the world. Our conclusion echoes J.D. Clark’s hypothesis ([119]: 252): “*MSA populations began to assume broad*, *regional identities during the time of the Last Interglacial (oxygen-isotope Stage 5*, *128*,*000–75*,*000 B*.*P*.*) and to produce tool-kits in part*, *at least*, *ecologically determined which*, *during the long dry period of lowered temperature during the earlier part of the Last glaciation (oxygen-isotope Stages 4 and 3*, *75*,*000–32*,*000)*, *began to show that the continuity was more with local antecedent occurrences than with contemporary assemblages in other regions*”.

The recognition of groups that were technologically distinct should however not be taken as rigid and impermeable cultural separations between them. Indeed, we observe a common technological background that transcends ecological biomes and that takes the form of the laminar blanks, which develop in southern Africa during the MIS5. In addition, we also notice the adoption of similar formal tools regardless of the technological and ecological contexts. This is the case for the bifacial pieces that flourish during the MIS 5 in South Africa [2,13,14,120]. This is additionally the case for the end-scrapers, which typify the Pietersburg phase ‘28’ at BRS but which are also recorded within the Still Bay phase of Blombos Cave and have been identified in the MSA 2 of Apollo 11.

Interestingly, behind these technological (the blades) and typological (the bifacial pieces, the end-scrapers) trends, we notice the existence of different traditions in the way blades are produced (see [2,117,121]), in the way bifacial pieces are shaped (see [2;11,13,14]) and in the way end-scrapers are manufactured. In that perspective, the present study provides a scenario where MIS5 groups were technologically distinct but connected, as would suggest the implementation of similar ideas but in different ways. The circulation of ideas between groups would indicate that MIS5 groups were related, a socio-economic context that has accompanied the set of innovations characterizing that time period.

The end-scrapers of the Pietersburg of BRS are the oldest expression of such tool types known to date. End-scrapers, considered as a typical tool of the Upper Palaeolithic package [122,123], introduce a new way of handling and using tools in order to scrape raw materials in a transversal motion. While end-scrapers represent a specialized tool in terms of motion, specimens from BRS -unlike recent examples strictly orientated towards the processing of hide (e.g., [103,112,124])- document the scraping of various raw materials such as bone, wood, minerals and hide. The appearance of end-scrapers reflects a change in the set of body motions and activities, which may be regarded as another level of complexity in terms of raw material processing and task organization during MIS5.

The southern African MSA provides alternative and complementary data to reconstitute the technological evolution that AMH societies experienced. In that perspective, the MIS5 represents a crucial period when many innovations were first expressed in the archaeological record. Based on technological comparisons, we propose that groups were regionally distinct but connected, encouraging future studies to build hypotheses following a cultural geographical approach (e.g., [125,126]). Clark [37,119] associated the beginning of the MSA with the beginning of regionalization. By attempting to understand under which circumstances modern human groups began to express themselves differently from each other, we are forced to address -at different scales of observation [127]- the nature of demographic and socio-economic boundaries.
